# METTL1 coordinates cutaneous squamous cell carcinoma progression via the m7G modification of the *ATF4* mRNA

**DOI:** 10.1038/s41420-025-02304-3

**Published:** 2025-01-27

**Authors:** Xinru Zhang, Tong Chen, Fanrong Zhang, Huanhuan Shi, Xiang Li, Zhijuan Wang, Dong Wang, Chao Hou

**Affiliations:** 1 School of Basic Medical Sciences, Anhui Medical University, Hefei, 230032 China; 2 The First Affiliated Hospital of Anhui Medical University, Hefei, 230022 China; 3 The Second Affiliated Hospital of Anhui Medical University, Hefei, 230032 China; 4 Inflammation and Immune Mediated Diseases Laboratory of Anhui Province, the Key Laboratory of Anti-inflammatory of Immune Medicines, Ministry of Education, Anhui Institute of Innovative Drugs, School of Pharmacy, Anhui Medical University, Hefei, 230032 China

**Keywords:** Squamous cell carcinoma, Epigenetics

## Abstract

Methyltransferase-like 1 (METTL1)-mediated m7G modification is a common occurrence in various RNA species, including mRNAs, tRNAs, rRNAs, and miRNAs. Recent evidence suggests that this modification is linked to the development of several cancers, making it a promising target for cancer therapy. However, the specific role of m7G modification in cutaneous squamous cell carcinoma (cSCC) is not well understood. In this study, we observed conspicuously elevated levels of METTL1 in cSCC tumors and cell lines. Inhibiting METTL1 led to reduced survival, migration, invasion, and xenograft tumor growth in cSCC cells. Mechanistically, through a combination of RNA sequencing, m7G methylated immunoprecipitation (MeRIP)-qPCR, and mRNA stability assays, we discovered that METTL1 is responsible for the m7G modification of *ATF4* mRNA, leading to increased expression of ATF4. Importantly, we demonstrated that this modification is dependent on the methyltransferase activity of METTL1. Additionally, we observed a positive association between ATF4 expression and METTL1 levels in cSCC tumors. Intriguingly, restoring ATF4 expression in cSCC cells not only promoted glycolysis but also reversed the anti-tumor effects of METTL1 knockdown. In conclusion, our results underscore the critical role of METTL1 and m7G modification in cSCC tumorigenesis, suggesting a promising target for future cSCC therapies.

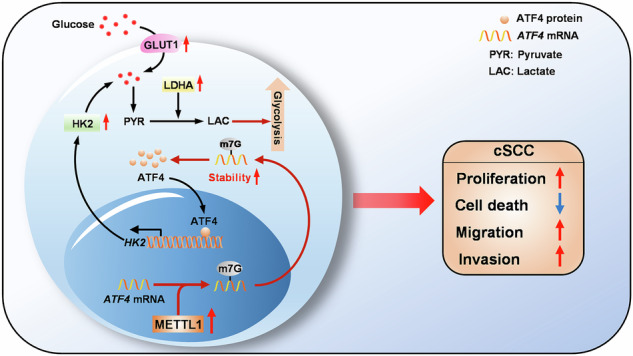

## Introduction

Skin cancer, which continues to be a highly destructive malignancy, is primarily attributed to DNA mutations or genetic defects that lead to unrepaired DNA in skin cells, resulting in the abnormal proliferation of these cells [1]. It is broadly categorized into melanoma and non-melanoma types, with the majority of cases being non-melanoma tumors originating from skin keratinocytes. Non-melanoma cases can be further classified into cutaneous basal cell carcinoma and squamous cell carcinoma (cSCC) of the skin [2]. cSCC, with its high glycolytic metabolism [3], is the second most prevalent form of skin cancer, accounting for approximately 20% of all skin malignancies, following closely behind basal cell carcinoma in terms of incidence. However, CSCC exhibits higher malignancy compared to basal cell carcinoma, with increased metastatic and invasive capabilities [4]. Current treatment options for CSCC include surgery, radiotherapy, chemotherapy, drug therapy, and targeted therapy. However, due to the inflammation and scarring resulting from surgery, radiotherapy, and chemotherapy, along with the physical and chemical characteristics of drugs and the limitations of skin barriers in the context of drug delivery or targeted therapy [5, 6], there remains an urgent need for more effective therapeutic strategies.

In the past few years, extensive research in RNA biology has revealed over 160 posttranscriptional RNA modifications. Among these modifications, N7-methylguanosine (m7G) modification was identified as a prevalent posttranscriptional RNA modification that affects tRNAs, rRNAs, miRNAs, as well as mRNAs, playing critical roles in RNA processing, metabolism, and functions [7]. The methyltransferase-like 1 (METTL1) enzyme is primarily responsible for catalyzing the methylation of m7G, with its co-factor WD repeat domain 4 (WDR4), helping to stabilize the METTL1/WDR4 complex [8]. Notably, METTL1/WDR4 has been found to catalyze the m7G modification of numerous internal mRNA sites. Subsequently, Quaking proteins selectively recognize these internal m7G-modified transcripts and facilitate their transport into stress granules by interacting with the core protein G3BP1, thereby enhancing mRNA stability and translation efficiency [9]. Emerging evidence has recently showcased the abnormal expression of METTL1 in tumor tissues and cell lines, underscoring the significant role of m7G modification in the development and progression of various cancers such as hepatocellular carcinoma (HCC) [10], head and neck squamous cell carcinoma (HNSCC) [11], oral squamous cell carcinoma [12] and osteosarcoma [13]. Therefore, METTL1 and m7G modification may potentially serve as biomarkers for early diagnosis and as targets for therapeutic interventions [14]. However, further investigation is needed to understand the differential expression of METTL1 in cSCC and the role of m7G modification in the development of this tumor type.

In this study, it was discovered that METTL1 is upregulated and plays an oncogenic role in cSCC. In vitro experiments showed that the survival, migration, and invasion of the cSCC cell lines HSC-1 and A431 were significantly inhibited upon METTL1 inhibition. Moreover, METTL1 knockdown weakened cSCC tumorigenesis in nude mice. Mechanistically, METTL1 was found to mediate the m7G modification of the *ATF4* mRNA, leading to its enhanced stability and subsequent expression through a mechanism dependent on METTL1’s methyltransferase activity. Furthermore, a positive correlation between ATF4 and METTL1 was observed in cSCC tumor tissues. Functionally, overexpressing ATF4 reversed the anti-tumor effect of METTL1 knockdown. These findings highlight the oncogenic role and mechanism of m7G modification by METTL1, offering potential therapeutic targets for cSCC.

## Results

### METTL1 is upregulated in cSCC tissues and cell lines

**Fig. 1:**
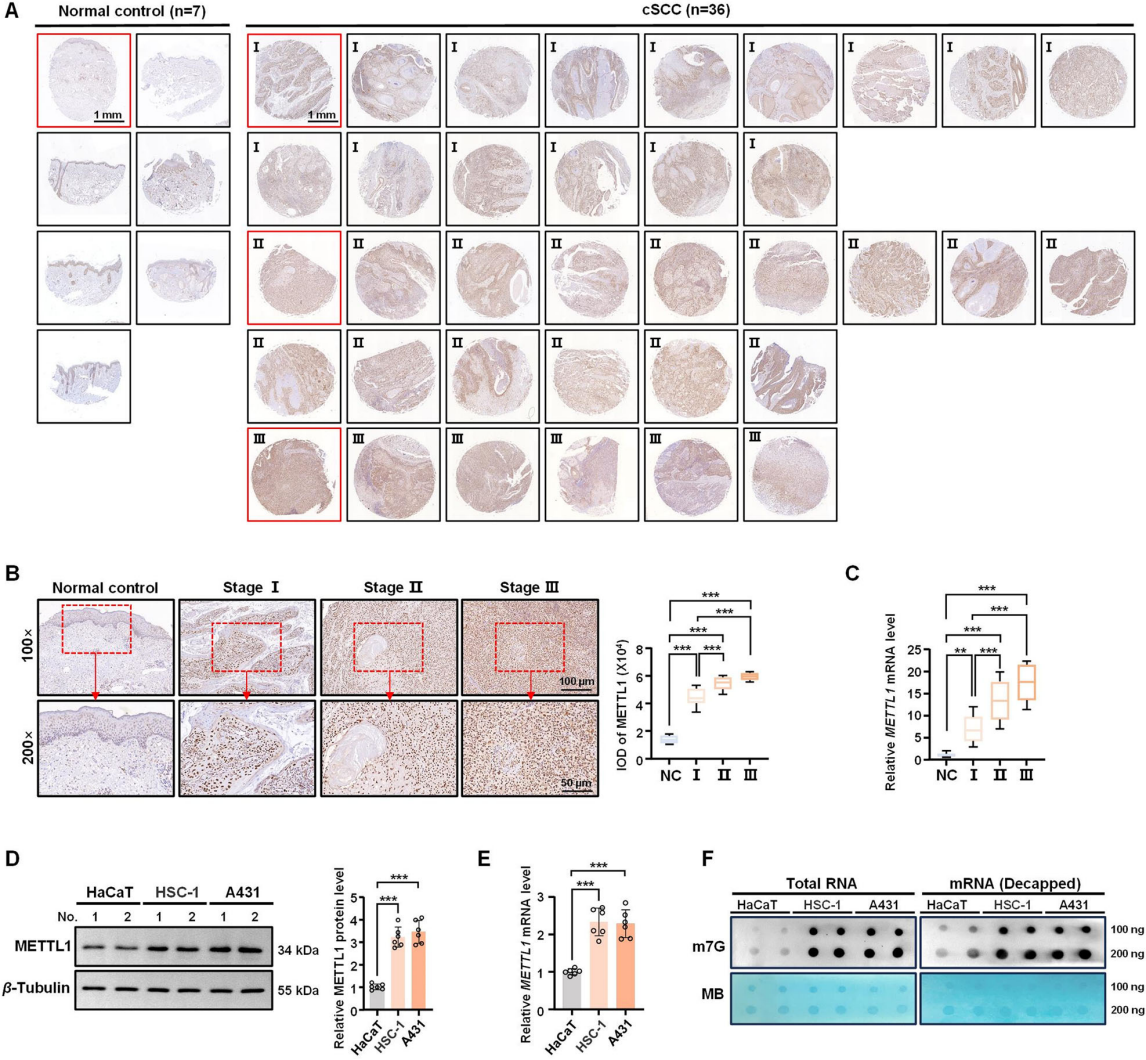
METTL1 is overexpressed in cSCC tumors and cell lines. **A** The protein levels of METTL1 in normal specimens (*n* = 7) and cSCC tumors (*n* = 36) with different tumor grade were detected by IHC. **B** The samples highlighted in red in **A** were magnified. The intensity optical density (IOD) for each sample was calculated. **C** The mRNA levels of *METTL1* in normal specimens and cSCC tumors were detected by qRT-PCR. **D**, **E** The expression of METTL1 in HaCaT keratinocytes and cSCC cell lines (HSC-1 and A431) were detected by western blot and qRT-PCR, respectively. *β*-Tubulin was used for the normalization control. The relative protein level of METTL1 was calculated. **F** The m7G methylation levels in total RNA and decapped mRNA from cell lines were detected by dot blot assay. Data are shown as mean ± SEM. ***P < 0.001, *n* = 6. MB methylene blue.

As a well-known methyltransferase, METTL1 is overexpressed in various tumor tissues and cell lines [15]. To determine the expression pattern of METTL1 in cSCC, we initially examined the protein level of METTL1 in paraffin-embedded sections from 36 cSCC and 7 normal specimens by IHC. METTL1 expression was significantly elevated in tumor tissues relative to normal skin tissues (Fig. 1A). Specifically, global METTL1 levels in tumor tissues continued to rise with increases in tumor stage (Fig. 1B). Subsequently, qRT-PCR was employed to assess the mRNA level of *METTL1* expression in tumor tissues, revealing a consistent increase in *METTL1* mRNA levels in cSCC compared to normal skin tissues (Fig. 1C). To further elucidate the potential involvement of METTL1 in tumor progression, we evaluated its protein and mRNA level along with m7G methylation levels in the cSCC cell lines (HSC-1 and A431) in comparison with control HaCaT keratinocytes. Markedly upregulated protein and mRNA levels of METTL1 were observed in HSC-1 and A431 cells relative to HaCaT controls (Fig. 1D, E). Importantly, dot blot results confirmed a significant increase in m7G methylation levels in total RNA and decapped mRNA of the cSCC cell lines (Fig. 1F). Taken together, our findings suggest that METTL1 is abnormally upregulated in cSCC tumors and cells, indicating its potential as a therapeutic target.

### METTL1 is crucial for cSCC cell survival

**Fig. 2:**
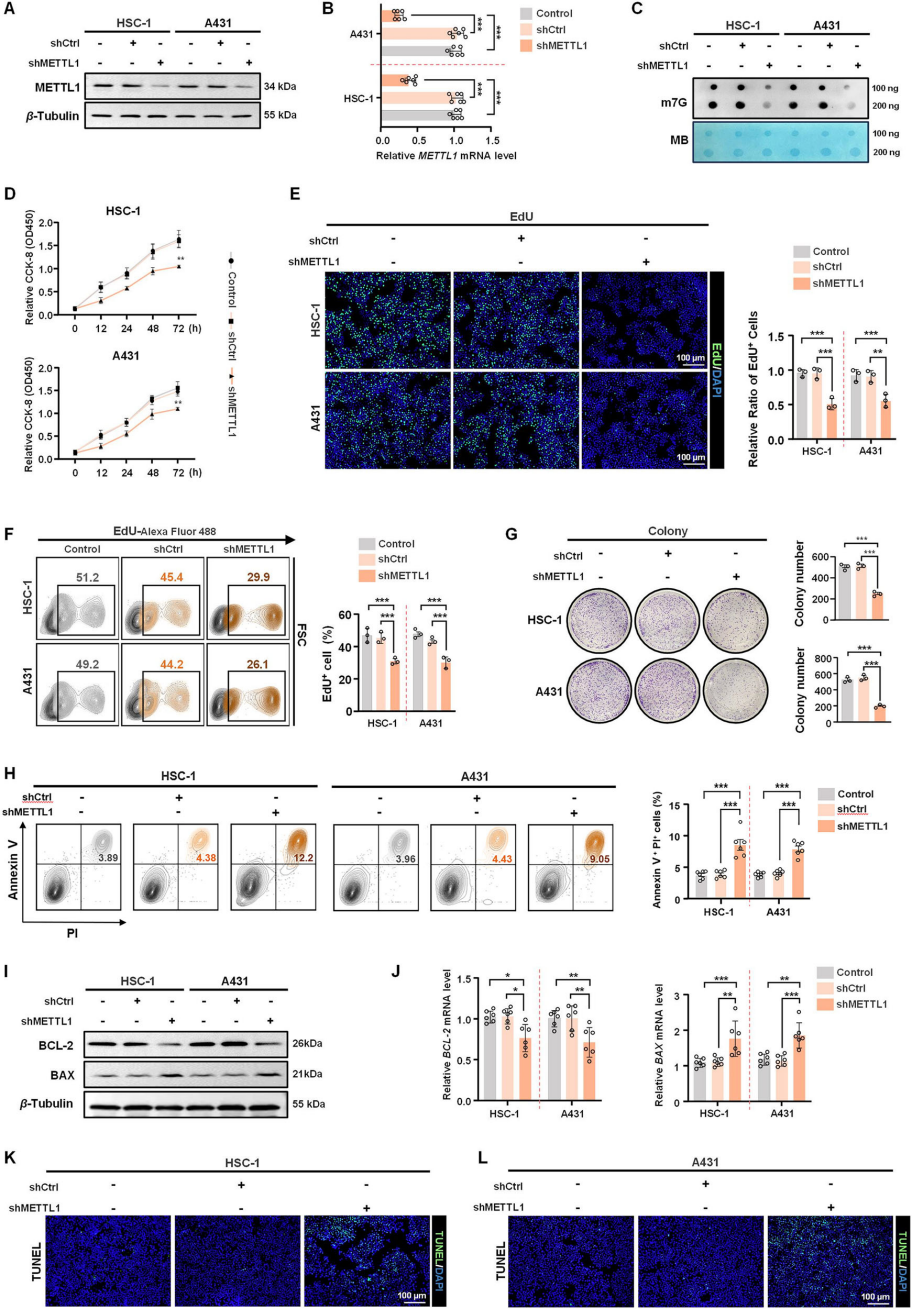
METTL1 knockdown significantly represses cSCC cells survival. **A**, **B** Western blot and qRT-PCR showed METTL1 was successfully knocked down by shMETTL1 at protein and mRNA levels. *β*-Tubulin was used for the normalization control. **C** The m7G methylation levels in METTL1-knockdown cSCC cell lines were detected by dot blot assay. **D** Measurements of cell viability by CCK8 assay (*n* = 6). **E** EdU staining assay was used to assess cell proliferation (*n* = 3). The relative ratio of EdU^+^ cells were calculated. **F** The proportions of EdU^+^ cells were detected by flow cytometry (*n* = 3). **G** Measurements of cell proliferation by colony formation assay (*n* = 3). The colony number was calculated. **H** The cell apoptosis was determined by Annexin V/PI double staining (*n* = 6). **I**, **J** The protein and mRNA levels of BCL-2 and BAX were detected by western blot and qRT-PCR (*n* = 6), respectively. *β*-Tubulin was used for the normalization control. **K**, **L** TUNEL staining of HSC-1 and A431 cells was performed (*n* = 3). Data are shown as mean ± SEM. *P < 0.05, **P < 0.01, ***P < 0.001.

To investigate the role of METTL1-mediated m7G modification in cSCC cell progression, we utilized an shRNA targeting *METTL1* to create stable METTL1 knockdown HSC-1 and A431 cell lines. The success of knockdown was confirmed based on decreased levels of *METTL1* mRNA and protein, with the most efficient knockdown being evident in cells being treated with shMETTL1-1 (Fig. 2A, B and Supplementary Fig. S1A–D). Notably, inhibition of METTL1 also led to a reduction in m7G methylation levels (Fig. 2C). Subsequent experiments were conducted to assess the impact of METTL1 knockdown on cSCC cell proliferation. A CCK8 assay revealed reduced HSC-1 and A431 cell viability after METTL1 knockdown (Fig. 2D). Cell proliferation was then assessed through an EdU assay, which showed a drastic decrease in EdU-positive cells upon METTL1 knockdown (Fig. 2E, F). These results were further supported by the colony formation assay, which revealed a lower number of colonies formed in the shMETTL1 group compared to the control group (Fig. 2G). These findings indicate that METTL1 plays a crucial role in promoting cSCC proliferation. Next, we sought to detect cSCC cell apoptosis following METTL1 knockdown. Annexin V /PI double staining indicated that METTL1 knockdown induced a reduction in the proportion of living cells and a simultaneous increase in the frequency of apoptosis (Fig. 2H). Furthermore, both the protein and mRNA levels of the anti-apoptotic factor BCL-2 were notably decreased upon METTL1 knockdown, while pro-apoptotic BAX levels were significantly elevated (Fig. 2I, J and Supplementary Fig. S2A). Consistent with these results, TUNEL staining further demonstrated that METTL1 deficiency promoted apoptosis in HSC-1 and A431 cells (Fig. 2K, L and Supplementary Fig. S2B). These findings underscore the pivotal role of METTL1 in cSCC cell survival.

### Inhibition of METTL1 suppresses the migration and invasion of cSCC cells

**Fig. 3:**
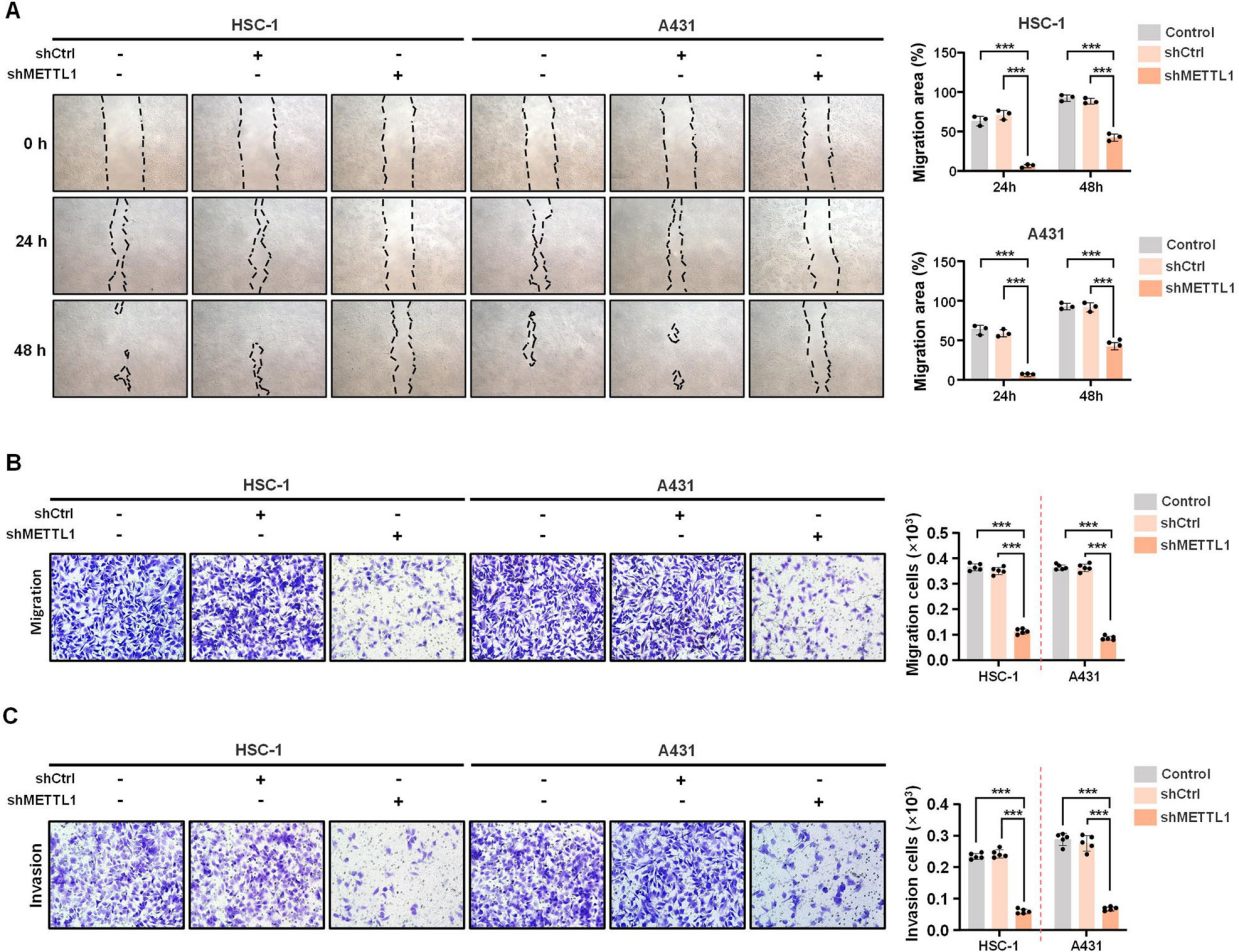
METTL1 knockdown inhibits migration and invasion of cSCC cells. **A** Migration of HSC-1 and A431 cells was evaluated by wound-healing assay (*n* = 3). The migration area was calculated. **B** The mobility was assessed by trans-well migration assay (*n* = 5). **C** The invasiveness of HSC-1 and A431 cells was evaluated by Matrigel invasiveness measurement (*n* = 5). Data are shown as mean ± SEM. ***P < 0.001.

Previous studies have demonstrated the ability of METTL1 to promote cancer cell migration and invasion [16]. To further validate these impacts of METTL1 on cSCC cells in vitro, wound healing, transwell assay, and matrigel invasion assay measurements were conducted. The results from the wound healing assay showed a larger wound area in the shMETTL1 group after 24 and 48 h compared to the control group (Fig. 3A). Additionally, the transwell migration assay revealed that METTL1 deficiency led to less efficient cell migration for both the HSC-1 and A431 cells (Fig. 3B). Furthermore, reduced invasiveness was observed in the shMETTL1 group (Fig. 3C). Collectively, these findings support the conclusion that inhibiting METTL1 suppresses cSCC cell survival, migration, and invasion.

### METTL1 is necessary for cSCC tumor growth in vivo

**Fig. 4:**
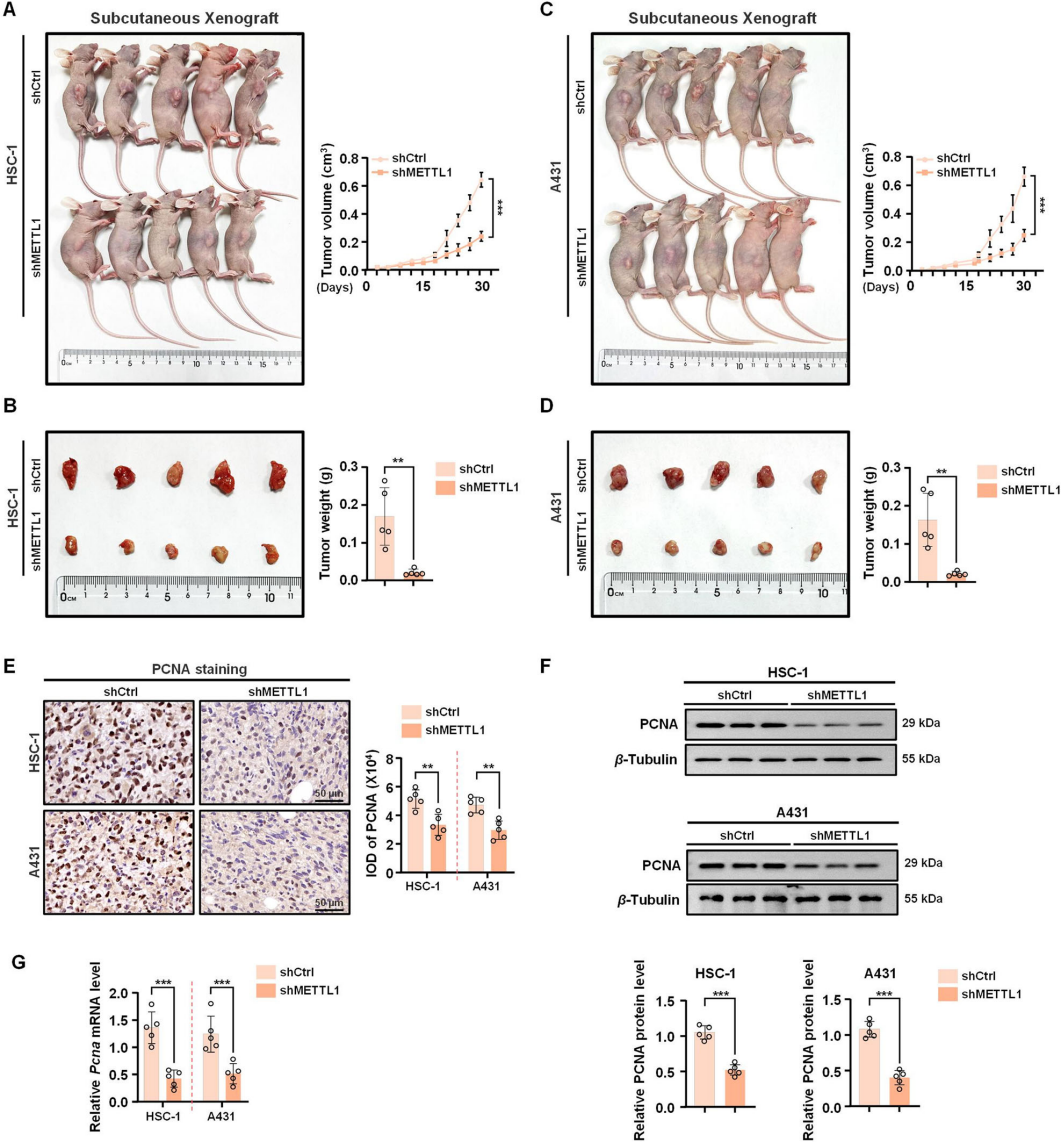
METTL1 deficiency reduces xenograft tumor growth in vivo. Knockdown of METTL1 inhibited HSC-1 xenograft tumor growth (**A**) and reduced tumor weight (**B**). Knockdown of METTL1 inhibited A431 xenograft tumor growth (**C**) and reduced tumor weight (**D**). **E**, **F** The protein levels of PCNA in tumor tissues were detected by IHC and western blot. The IOD of PCNA was calculated. *β*-Tubulin was used for the normalization control. The relative protein level was calculated. **G** The mRNA level of *Pcna* was evaluated by qRT-PCR. Data are shown as mean ± SEM. *n* = 5. **P < 0.01, ***P < 0.001.

To comprehensively investigate the in vivo functional role of METTL1, a xenograft tumor model was established by subcutaneously transplanting mice with HSC-1 and A431 cells transfected with either shCtrl or shMETTL1. We initially found that the protein levels of METTL1 in the tumor tissues originating from METTL1-knockdown cells were significantly decreased than those originating from control cells (Supplementary Fig. S3A, B). Importantly, tumors in nude mice injected with METTL1-knockdown cells exhibited reduced growth rates compared to the control group (Fig. 4A, C). Moreover, the final xenograft weight was notably lower in the shMETTL1 groups (Fig. 4B, D). Subsequent detection of PCNA levels in tumor tissues revealed decreased protein and mRNA expression in the shMETTL1 group, as indicated by IHC, western blotting, and qRT-PCR analyses (Fig. 4E–G). Collectively, these findings support the conclusion that METTL1 functions as an oncogene in cSCC.

### METTL1 mediates the m7G methylation modification of the *ATF4* mRNA

**Fig. 5:**
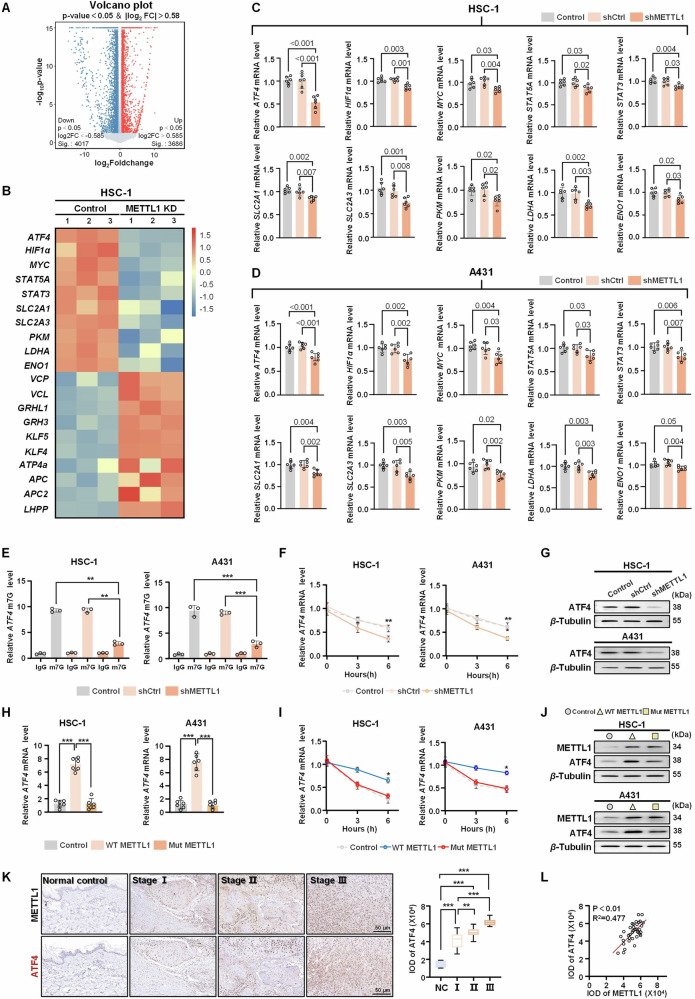
METTL1 induces the m7G modification of *ATF4* mRNA. **A** Volcano plot of significantly altered mRNA in METTL1-knockdown HSC-1 cell compared to control cell. **B** Differentially expressed genes (DEG) were clustered and shown in a heat map of RNA sequencing. **C**, **D** qRT-PCR was performed to detect the downregulated DEG in HSC-1 and A431 cells (*n* = 6). **E** Suppressed m7G methylated *ATF4* mRNA in METTL1-knockdown cells was detected by MeRIP-qPCR (*n* = 3). **F** qRT-PCR showing *ATF4* mRNA transcripts stability in ActD-treated HSC-1 and A431 cells transfected with or without shMETTL1 (*n* = 3). **G** The protein levels of ATF4 in HSC-1 and A431 cells with or without shMETTL1 were detected by western blot (*n* = 3). *β*-Tubulin was used for the normalization control. **H** The mRNA levels of *ATF4* in cells transfected with WT METTL1 or Mut METTL1 were assessed by qRT-PCR (*n* = 6). **I** qRT-PCR showing *ATF4* mRNA transcripts stability in ActD-treated HSC-1 and A431 cells transfected with WT METTL1 or Mut METTL1 (*n* = 3). **J** The protein levels of ATF4 in cells transfected with WT METTL1 or Mut METTL1 were assessed by western blot (*n* = 3). *β*-Tubulin was used for the normalization control. **K** The protein levels of METTL1 and ATF4 in normal specimens (*n* = 7) and cSCC tumors (*n* = 36) with different tumor grade were detected by IHC on continuous sections. The intensity optical density (IOD) for each sample was calculated. **L** Scatter plots showing the positive correlation between METTL1 and ATF4 expression in cSCC tumors. Data are shown as mean ± SEM. *P < 0.05, **P < 0.01, ***P < 0.001.

To systematically explore the mechanism through which METTL1 promotes cSCC tumorigenesis, we conducted bulk RNA-seq analyses to identify differentially expressed genes (DEGs) in METTL1-knockdown and control cells. Knockdown of METTL1 in HSC-1 cells resulted in the downregulation of 4017 genes and upregulation of 3686 genes (Fig. 5A). Previous studies have shown that METTL1-mediated m7G modification enhances mRNA stability, leading to increased expression and subsequent transcription [17]. Therefore, our focus was on the genes that were downregulated following METTL1 knockdown. Of these genes, we observed a significant decrease in glycolytic metabolism-related genes in METTL1-knockdown HSC-1 cells, including *ATF4*, *HIF1α*, *MYC*, *STAT5A*, *STAT3*, *SLC2A1*, *SLC2A3*, *PKM*, *LDHA*, and *ENO1* (Fig. 5B). Subsequently, qRT-PCR assays were performed to validate the heat map results from the HSC-1 and A431 cells transfected with shCtrl or shMETTL1. These analyses confirmed that all of these DEGs were significantly downregulated after METTL1 knockdown (Fig. 5C, D). To identify potential mRNAs that can be modified and regulated by METTL1, a m7G-RIP-qPCR analysis was performed in HSC-1 and A431 cells. The knockdown of METTL1 led to a decrease in the m7G enrichment of the *ATF4* mRNA in both cell lines, while other genes remained unaffected (Fig. 5E and Supplementary Fig. S4A, B). Furthermore, we observed a marked decrease in the m7G level of decapped *ATF4* mRNA following METTL1 deficiency (Supplementary Fig. S4C), suggesting that internal *ATF4* mRNA may be a target for m7G methylation. Interestingly, m7G-MeRIP-seq data from a separate study strongly indicated the presence of potential internal m7G sites in *ATF4* mRNA of HepG2 and HeLa cells [18]. Combined with this study and the updated database for decoding the m7G epitranscriptome (http://www.rnamd.org/m7GHub2/index.html), we aimed to further confirm that METTL1 targets ATF4 mRNA for m7G modifications. Specifically, we predicted that “TCAGGCT” in the 5’UTR may represent a potential m7G site for ATF4 mRNA. Subsequently, we established *ATF4* m7G motif mutants by replacing the guanine (G) in the fourth position with cytosine (C). The dual-luciferase assay demonstrated that the wild-type (WT) ATF4 reporter exhibited a decrease upon METTL1 knockdown, while no significant impact was observed after transfections with the mutant ATF4 (Supplementary Fig. S4D). Next, HSC-1 and A431 cells were treated with actinomycin D, a transcriptional inhibitor, and the half-life of the *ATF4* transcript was detected. As expected, we detected decreased mRNA stability in the shMETTL1 group (Fig. 5F). Consistent with these mRNA levels, the protein level of ATF4 was also found to be reduced in the shMETTL1 group (Fig. 5G). To further verify whether the effect of METTL1 on mRNA stability is dependent on its methyltransferase activity, we introduced WT and catalytically inactive METTL1 (amino acids 160–163 changed from LFPD to AFPA, hereafter referred to as Mut METTL1) plasmids, as previously reported [19], into HSC-1 and A431 cells. Dot blot results revealed that the overexpression of WT METTL1 significantly elevated m7G methylation levels, while the overexpression of Mut METTL1 did not (Supplementary Fig. S5A, B). Functionally, WT METTL1 overexpression notably enhanced the levels and stability of the *ATF4* mRNA, while Mut METTL1 overexpression had no significant impact on the *ATF4* mRNA (Fig. 5H, I). Furthermore, transfection with WT METTL1 led to an increase in ATF4 protein levels, whereas transfection with Mut METTL1 did not show the same effect (Fig. 5J). Representative images of IHC staining for ATF4 showed a significant increase in ATF4 levels in cSCC tumor tissues (Fig. 5K). Importantly, there was a positive correlation between ATF4 and METTL1 at the protein level (Fig. 5L). Overall, these gain-/loss-of-function experiments suggest that METTL1 mediates m7G modification of *ATF4* mRNA to enhance its stability and subsequent expression, with these effects depending on the enzyme activity of METTL1.

### ATF4 rescue drives metabolic reprogramming to promote cSCC progression

**Fig. 6:**
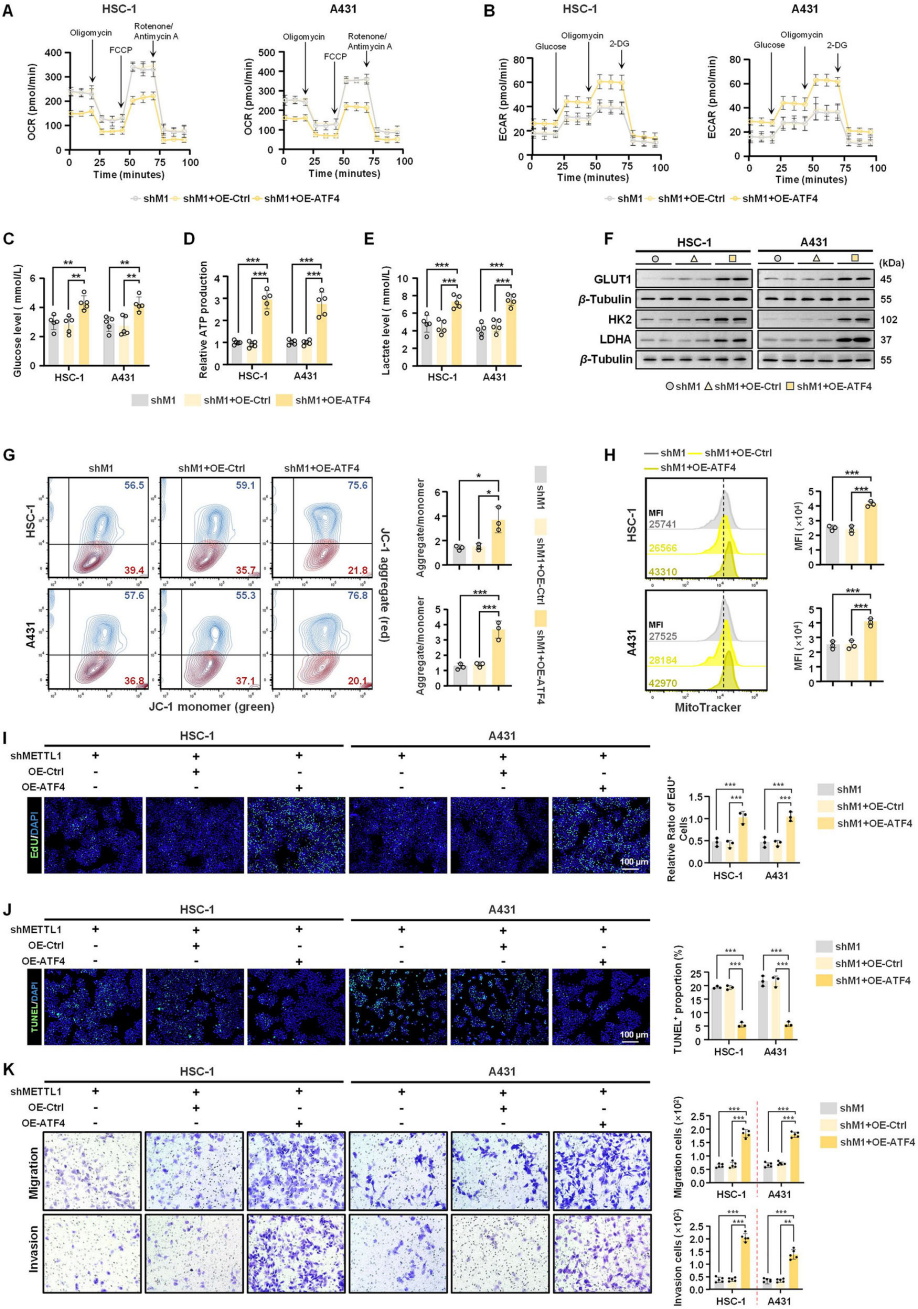
ATF4 overexpression reverses the anti-tumor effect of METTL knockdown. **A**, **B** Metabolic phenotypes of HSC-1 and A431 cells were determined by the both OCR and ECAR assays (*n* = 5). **C** Glucose uptake in HSC-1 and A431 cells were detected glucose assay (*n* = 5). **D** The ATP production was evaluated through ATP assay kit (*n* = 5). **E** Lactate levels in the medium were assessed (*n* = 5). **F** The protein levels of GLUT1, HK2 and LDHA were detected by western blot (*n* = 5). *β*-Tubulin was used for the normalization control. **G** MMP was measured by flow cytometry (*n* = 3). **H** The mitochondrial mass was measured by MitoTracker (*n* = 3). **I** EdU staining assay was used to assess cell proliferation (*n* = 3). **J** TUNEL staining of HSC-1 and A431 cells was performed (*n* = 3). **K** The mobility and invasiveness were assessed by trans-well migration assay and matrigel invasiveness measurement (*n* = 5), respectively. Data are shown as mean ± SEM. *P < 0.05, ***P < 0.001.

To further probe whether the impact of METTL1 on tumorigenesis is influenced by ATF4, ATF4 plasmids were transfected into METTL1-knockdown HSC-1 and A431 cells to induce the overexpression of ATF4 (Supplementary Fig. S7A and Table 2). Interestingly, the culture medium of cells overexpressing ATF4 turned yellow more rapidly after 24 h, indicating a potential increase in nutrient consumption by these cells (Supplementary Fig. S7B). ATF4 plays a crucial role in metabolic remodeling by enhancing glycolysis through direct binding to the promoter of *HK2*, leading to increased HK2 expression [20]. To study the metabolic changes resulting from ATF4-overexpression, mitochondrial function was assessed using the Seahorse XF96 extracellular flux analyzer. The results showed a significant decrease in OCR across various parameters such as basal, ATP production, maximal respiration, and spare respiratory capacity, accompanied by an increase in ECAR including glycolysis, glycolytic capacity, and reserve capacity (Fig. 6A, B and Supplementary Fig. S7C–F). Additionally, glucose consumption, ATP production, and lactate concentration all increased following ATF4 rescue (Fig. 6C–E). Next, several key factors involved in metabolic progression were identified. Glucose transporter protein-1 (GLUT-1) facilitates the cellular uptake of glucose, leading to increased glucose consumption by tumor cells to drive metabolic reprogramming [21]. Hexokinase2 (HK2) serves as the initial rate-limiting enzyme in glycolysis and is commonly upregulated in tumors with heightened aerobic glycolysis [22]. Lactate dehydrogenase A (LDHA) plays a crucial role in converting pyruvate to lactate in tumor lactate metabolism [23]. Notably, all of these proteins were significantly upregulated in ATF4-overexpressing HSC-1 and A431 cells compared to control cells (Fig. 6F). We then investigated whether METTL1 deficiency itself also influenced the metabolic phenotype. Intriguingly, all of the aforementioned indicators indicated that the disruption of METTL1 inhibited glycolysis in HSC-1 and A431 cells (Supplementary Fig. S6A–G). To further investigate whether the discrepancy in ATF4 expression mediated by METTL1 might regulate the transcriptional activation of *HK2*, luciferase activity of *HK2* was assessed in HSC-1 and A431 cells both with and without METTL1 overexpression and ATF4 knockdown. The results indicated that luciferase activity of *HK2* was significantly enhanced following METTL1 overexpression; however, a marked decrease was observed upon ATF4 knockdown, even in the presence of METTL1 overexpression (Supplementary Fig. S7G), indicating that METTL1 could regulate the promoter of *HK2* depending on ATF4. These findings suggest that ATF4 rescue may enhance glycolytic activity in METTL1-knockdown HSC-1 and A431 cells.

Tumor cells require ATP generated through glycolysis in the cytoplasm, but maintaining mitochondrial homeostasis and increasing the overall mitochondrial mass are also crucial for cell growth in response to changing microenvironmental stressors [24]. Mitochondrial membrane potential (MMP), a critical indicator, is indicative of electron transport and oxidative phosphorylation activities, which are essential for ATP production in various biological processes and for the development of human cancer [25]. JC-1 can form red fluorescent aggregates in healthy mitochondria, but is transformed to green fluorescent monomers in depolarized mitochondria. The ratio of JC-1 red/green fluorescence can serve as an index for MMP and can also reflect early cell apoptosis. As shown in Fig. 6G, ATF4 rescue significantly increased the proportions of aggregate, indicating the increased MMP. Consistently, ATF4-overexpressing HSC-1 and A431 cells exhibited higher mitochondrial mass, as indicated using the MitoTracker dye, compared to METTL1-knockdown cells (Fig. 6H). Subsequently, we aimed to investigate the pathological implications of ATF4 overexpression in METTL1-knockdown HSC-1 and A431 cells. Cell survival was assessed using EdU and TUNEL staining assays, revealing that ATF4 rescue reversed the inhibitory effects of shMETTL1 on cell growth, characterized by an increase in EdU-positive cells and a decrease in TUNEL-positive cells (Fig. 6I, J). Furthermore, forced expression of ATF4 in METTL1-knockdown cells led to enhanced cell migration and invasion (Fig. 6K). Overall, our results suggest that METTL1-mediated cSCC cell growth is facilitated by the ATF4-induced promotion of glycolysis.

### Upregulation of ATF4 blocks METTL1 silencing-induced anti-tumor effects in cSCC cells

**Fig. 7:**
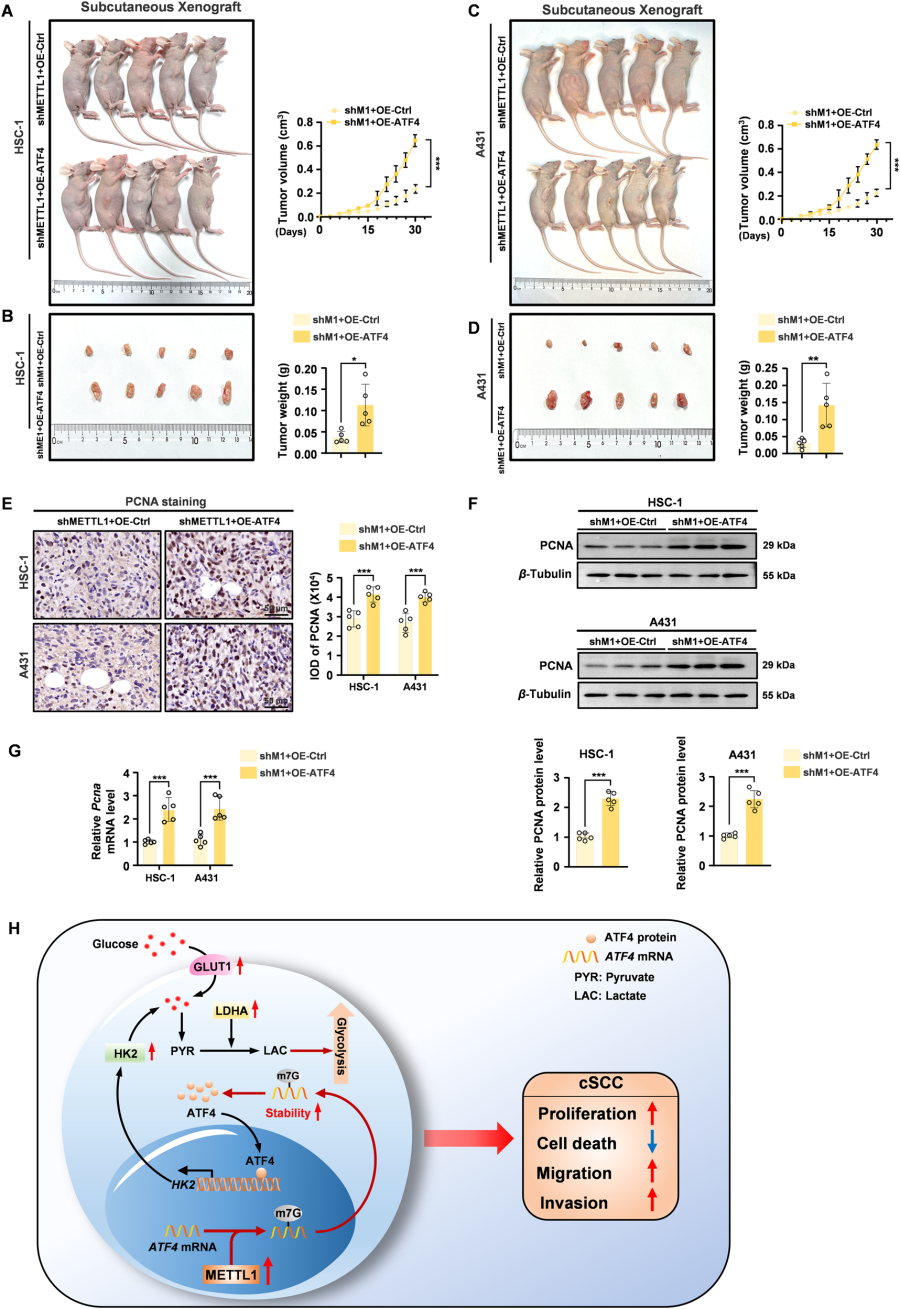
ATF4 overexpression counteracted the inhibitory effects of METTL knockdown on tumor growth in vivo. ATF4 overexpression promoted METTL1-deficient HSC-1 xenograft tumor growth (**A**) and reduced tumor weight (**B**). ATF4 overexpression promoted METTL1-deficient A431 xenograft tumor growth (**C**) and reduced tumor weight (**D**). **E**, **F** The protein levels of PCNA in tumor tissues were detected by IHC and western blot. The IOD of PCNA was calculated. *β*-Tubulin was used for the normalization control. The relative protein level was calculated. **G** The mRNA level of *Pcna* was evaluated by qRT-PCR. **H** The schematic diagram of oncogenic effects of METTL1-midiated m7G modification in cSCC through enhancing the mRNA stability of *ATF4* and subsequently expression. Data are shown as mean ± SEM. *n* = 5. **P < 0.01, ***P < 0.001.

Next, we further evaluated whether ATF4 overexpression could diminish the therapeutic efficacy of METTL1 inhibition. Subcutaneous injections of METTL1-knockdown HSC-1 and A431 cells transfected with either a vector control construct or an ATF4-overexpression plasmid were administered to nude mice. Initially, the expression levels of METTL1 and ATF4 in the tumor tissues were examined, revealing a significant enhancement in ATF4 expression in tumors originating from cells harboring the overexpression of ATF4 (Supplementary Fig. S8A–D). ATF4 overexpression mitigated the impact of METTL1 knockdown on tumor growth and weight (Fig. 7A–D). Furthermore, we observed that ATF4 overexpression increased the proliferation of cSCC cells in vivo, as evidenced by elevated levels of PCNA protein (Fig. 7E, F) and mRNA (Fig. 7G). In conclusion, these findings provide further evidence that METTL1 promotes cSCC tumorigenesis by upregulating ATF4 expression.

## Discussion

As a high-grade skin cancer, there have been multiple attempts by researchers to develop treatments for cSCC, but these efforts have thus far been unsuccessful. Our data established METTL1 as an oncogenic factor in cSCC, making it a promising target for treatment (Fig. 7H). Here, we found that METTL1 was gradually upregulated in cSCC tumors and cell lines and was significantly associated with clinical progression. With the loss of METTL1 expression in vitro, cSCC cell lines failed to proliferate, migrate, and invade. Additionally, in vivo experiments demonstrated that tumor growth following HSC-1 and A431 cell implantation was suppressed upon METTL1 deletion. Mechanistically, we confirmed that METTL1 could induce the m7G modification of the *ATF4* mRNA to enhance its stability. Importantly, consistent protein expression patterns for ATF4 and METTL1 were observed in cSCC tumors. The upregulation of ATF4 in cSCC cells counteracted the anti-tumor effect of METTL1 knockdown by enhancing glycolytic capability in cSCC cells. Overall, our study underscores the significance of METTL1 and its role in the m7G modification of *ATF4* as a means of regulating cSCC tumor progression, offering a foundation for the further exploration of innovative therapeutic approaches to clinical cSCC treatment.

Cellular RNAs can undergo various chemical modifications that play crucial roles in RNA metabolism, contributing to the intricate process of gene regulation. This has led to the exploration of a new field known as ‘RNA epigenetics’. One important form of RNA modification is the m7G modification, catalyzed by METTL1, with WDR4 acting as a co-factor to stabilize the METTL1/WDR4 complex. The significance of m7G modification in tumor development makes it a particularly compelling area of study within the realm of RNA epigenetics [26]. In patients with HCC, elevated METTL1 expression is closely linked to advanced tumor stage and poor clinical outcomes [27]. Another study has demonstrated that inhibiting METTL1 and WDR4 globally can suppress m7G tRNA modification, resulting in hindered HCC progression [28]. Moreover, increased METTL1 levels have been associated with an immunosuppressive microenvironment in recurrent HCC by enhancing TGF-*β*2 translation [29]. Similarly, upregulated METTL1 and WDR4 have been identified in HNSCC and correlated with a negative prognosis. METTL1 deficiency in tumor cells inhibits the m7G modification of 16 tRNAs, leading to a reduction in the global translation of oncogenic transcripts and subsequently inhibiting HNSCC progression [11]. Additionally, research by Xie et al. has revealed that METTL1 promotes the m7G modification of miR-760 to expedite the degradation of the *ATF3* mRNA, thereby accelerating bladder cancer growth. Interestingly, decreased levels of METTL1 have been observed in osteosarcoma, with low METTL1 expression playing an oncogenic role through the regulation of ferroptosis mediated by the pri-miR-26a/FTH1 axis [30]. Overall, these studies confirm that while METTL1 expression may vary across different tumor types, it consistently regulates tumor growth. Increased expression of METTL1 is crucial in promoting cSCC growth both in vivo and in vitro, suggesting that targeting METTL1 could be a promising strategy for cSCC treatment. However, further validation is needed to determine the expression and impact of METTL1 on cutaneous basal cell carcinoma, which is the most common skin cancer and is characterized by relatively low levels of malignancy.

Metabolic adaptation toward aerobic glycolysis, known as the Warburg effect, is a defining characteristic of cancer. This shift is driven by the heightened need for ATP in rapidly dividing tumor cells, resulting in elevated concentrations of lactate both inside and outside the cells compared to cells at rest [31]. In our study, we observed a significant decrease in the mRNA levels of glycolytic metabolism-related genes following METTL1 knockdown. We therefore explored the specific genes that undergo direct m7G modification, as well as the potential direct involvement of m7G in the regulation of glycolysis. Importantly, a series of experiments led us to conclusively identify the *ATF4* mRNA as the primary target for METTL1-mediated m7G modification. It has been documented that an increase in ATF4 levels, triggered by the downregulation of the mitochondrial electron transport chain in *Drosophila* eye progenitor cells, led to heightened proliferation by inducing a Warburg-like phenotype [32]. In macrophages, ATF4 has been identified as a key glycolytic activator that contributes to the inflammatory response in sepsis by binding to the promoter region of *Hk2* [20]. Furthermore, Zhang et al. found that ATF4-mediated expression of phosphoglycerate dehydrogenase enhances glycolysis in tumor-associated endothelial cells, resulting in endothelial cell overgrowth and ultimately promoting glioblastoma resistance to immunotherapy [33]. Moreover, heightened ATF4 expression is crucial for tumor cell growth and acts as a defense mechanism against tumor cell death [34]. These findings underscore the significance of ATF4 as a critical glycolytic activator in both tumorigenesis and immune responses. Our results align well with these studies, showing that the overexpression of ATF4 significantly boosts glycolysis in METTL1-knockdown cSCC cells, indicating a connection between m7G modification and tumor glycolysis. Furthermore, we observed alterations in other key metabolic indicators in METTL1-knockdown cSCC cells following ATF4 overexpression, including GLUT1, HK2, and LDHA. GLUT1, a member of the GLUT family, plays a crucial role in initiating glucose utilization within cells. In various malignancies, the rate of glucose uptake is positively associated with GLUT expression, particularly GLUT1 [35]. Hexokinase, specifically HK2, is a pivotal enzyme in glucose metabolism regulation and energy metabolism in tumor cells [36]. Increased expression of HK2 has been identified in several cancer types, such as melanoma [37], glioma [38], and bladder cancer [39], impacting both glycolysis and mitochondrial function. Additionally, LDHA is a critical enzyme involved in the conversion of pyruvate to lactate [40]. Notably, all of these metabolic indicators were upregulated in METTL1-deficient HSC-1 and A431 cells following ATF4 overexpression, further supporting the role of METTL1 in promoting tumorigenesis through ATF4-mediated glycolysis.

Recently, the disruption of mitochondrial homeostasis, such as through efforts to reduce mitochondrial MMP and mass, has emerged as a potential therapeutic approach for cancer treatment [41–43]. Our findings highlight the significant role of ATF4 in preventing mitochondrial depolarization and decreasing mitochondrial mass, as demonstrated by JC-1 and mitoTraker assays in cSCC cells lacking METTL1. This raises the question of why ATF4 rescue led to an improvement in mitochondrial homeostasis while also reducing oxygen consumption (lower OCR). This may be attributed to the significant role of mitochondrial metabolism in tumor growth. In addition to generating ATP through the tricarboxylic acid (TCA) cycle, the intermediate metabolites of the TCA cycle are essential for tumor cell proliferation and the production of oncometabolites that support tumorigenesis rather than ATP production. Therefore, the relationship between restored mitochondrial homeostasis and increased oxygen consumption in tumors is not direct [44]. The seemingly conflicting role of ATF4 overexpression in METTL1-knockdown HSC-1 and A431 cells can be further clarified by examining the impact of mitochondrial metabolism in cSCC. In summary, there is a need for further exploration of the significance of METTL1-mediated m7G modification in the context of cutaneous cancer metabolism.

## Materials and methods

### Patient samples

A tissue array (HSkiC060PT01) containing 36 cSCC specimens and 7 normal cutaneous specimens was obtained from Shanghai Outdo Biotech Co., Ltd. Tumors were classified according to the cSCC Broders Pathological Classification. There were 15, 15, and 6 patients with stage I, II, and III disease, respectively. Clinicopathological parameters such as age, sex, pathological grading, size, and infiltration were retrieved from patients’ medical records (Table 1). Fresh samples collected during surgery were immediately frozen in liquid nitrogen for subsequent total RNA extraction. Written informed consent was obtained from all patients for the use of surgical samples. All experiments involving human tissues were carried out in compliance with the Helsinki Declaration and were approved by the Institutional Review Board of Shanghai Outdo Biotech Co., Ltd. (approval number SHYJS-CP-2001009).

**Table 1:** Basic information for healthy people and cSCC patients.

Number	Sex	Age (year)	Pathological type	Pathological grading	Tumor site	Degree of infiltration
1	Male	71	SCC	I	Tempus	N/A
2	Male	77	SCC	I	Left auricle	N/A
3	Male	35	SCC	I	Left foot root	N/A
4	Male	54	SCC	I	Scalp	N/A
5	Male	70	SCC	I	Right cheek	Dermis
6	Male	63	SCC	I	Bottom of left foot	N/A
7	Male	67	SCC	I	Left tempus	N/A
8	Male	58	SCC	I	Scalp	Dermis
9	Female	50	SCC	I	Scalp	N/A
10	Female	75	SCC	I	Left Tempus	N/A
11	Female	91	SCC	I	Left frontal face	Striated muscle
12	Female	76	SCC	I	Left Tempus	N/A
13	Male	73	SCC	I	Left Tempus	Subcutaneous tissue
14	Male	74	SCC	I	Auricle	N/A
15	Male	78	SCC	I	Vulva	Subcutaneous tissue
16	Female	83	SCC	II	Right cheek	Superficial muscular layer
17	Female	86	SCC	II	Left forearm	N/A
18	Male	82	SCC	II	Right eye	N/A
19	Male	74	SCC	II	Skin	N/A
20	Female	39	SCC	II	Scalp	Subcutaneous tissue
21	Male	70	SCC	II	Left cheek	Dermal reticular layer
22	Male	76	SCC	II	Occipital posterior	N/A
23	Female	81	SCC	II	Back of the right hand	N/A
24	Male	82	SCC	II	Right tempus	Dermal papilla layer
25	Male	83	SCC	II	Left cheek	Dermis
26	Female	84	SCC	II	Left wrist	N/A
27	Female	74	SCC	II	Right cheek	Subcutaneous adipose tissue
28	Female	89	SCC	II	Left cheek	Dermal reticular layer
29	Male	95	SCC	II	Left frontal face	N/A
30	Female	68	SCC	II	Eternal auditory canal	N/A
31	Female	86	SCC	III	Right thigh	Dermis and Subcutaneous adipose tissue
32	Female	95	SCC	III	Left tmpus	N/A
33	Female	43	SCC	III	Posterior wall	Full layer
34	Male	79	SCC	III	Right auricle	N/A
35	Female	36	SCC	III	Left nose	N/A
36	Female	74	SCC	III	Right cheek	N/A
37	Male	adult	N/A	N/A	N/A	N/A
38	Male	adult	N/A	N/A	N/A	N/A
39	Female	adult	N/A	N/A	N/A	N/A
40	Female	adult	N/A	N/A	N/A	N/A
41	Female	adult	N/A	N/A	N/A	N/A
42	Male	adult	N/A	N/A	N/A	N/A
43	Male	adult	N/A	N/A	N/A	N/A

### Cell lines and cell culture

The cSCC cell lines HSC-1, A431, and the human benign epidermal keratinocyte HaCaT cell line were procured from Procell (Wuhan, China). These cell lines were cultured in Dulbecco’s Modified Eagle’s Medium (DMEM, Gibco, USA) supplemented with 10% fetal bovine serum (FBS, ThermoFisher, USA) and 1% penicillin-streptomycin. The cells were maintained at 37 °C in a 5% CO_2_ atmosphere.

### Lentivirus infection and cell transfection

HSC-1 and A431 cells in the logarithmic growth phase were transfected with a lentivirus-mediated shRNA targeting human METTL1 (shMETTL1) obtained from Genco Biotechnology (Beijing, China) to knock down METTL1 expression. Stably transformed cell lines were selected and maintained with puromycin (1 μg/mL). The sequence of shMETTL1 is provided in Table 2. Plasmids expressing wild-type METTL1 and a catalytically dead mutant (amino acids 160–163 changed from LFPD to AFPA) were used for METTL1 overexpression, as previously described [19].

**Table 2:** The sequences for shRNA.

Name	Sequence
*shMETTL1-1*	GGTGTATACCATAACCGATGT
*shMETTL1-2*	CCCACATTTCAAGCGGACAAA
*shMETTL1-3*	GATGACCCAAAGGATAAGAAA
The sequences of primers used for RT-qPCR (human)	
*METTL1* F	5’-GGCAACGTGCTCACTCCAA-3’
*METTL1* R	5’-CACAGCCTATGTCTGCAAACT-3’
*BCL-2* F	5’-GGTGGGGTCATGTGTGTGG-3’
*BCL-2* R	5’-CGGTTCAGGTACTCAGTCATCC-3’
*BAX* F	5’-CCCGAGAGGTCTTTTTCCGAG-3’
*BAX* R	5’-CCAGCCCATGATGGTTCTGAT-3’
*ATF4* F	5’-ATGACCGAAATGAGCTTCCTG-3’
*ATF4* R	5’-GCTGGAGAACCCATGAGGT-3’
*HIF1α* F	5’-GAACGTCGAAAAGAAAAGTCTCG-3'
*HIF1α* R	5’-CCTTATCAAGATGCGAACTCACA-3’
*MYC* F	5’-GGCTCCTGGCAAAAGGTCA-3’
*MYC* R	5’-CTGCGTAGTTGTGCTGATGT-3’
*STAT5A* F	5’- GCAGAGTCCGTGACAGAGG-3’
*STAT5A* R	5’-CCACAGGTAGGGACAGAGTCT-3’
*STAT3* F	5’-CAGCAGCTTGACACACGGTA-3’
*STAT3* R	5’-AAACACCAAAGTGGCATGTGA-3’
*SLC2A1* F	5’-ATTGGCTCCGGTATCGTCAAC-3’
*SLC2A1* R	5’-GCTCAGATAGGACATCCAGGGTA-3'
*SLC2A3* F	5’- GCTGGGCATCGTTGTTGGA-3’
*SLC2A3* R	5’- GCACTTTGTAGGATAGCAGGAAG-3’
*PKM* F	5’-ATGTCGAAGCCCCATAGTGAA-3’
*PKM* R	5’-TGGGTGGTGAATCAATGTCCA-3’
*LDHA* F	5’-TTGACCTACGTGGCTTGGAAG-3’
*LDHA* R	5’-GGTAACGGAATCGGGCTGAAT-3’
*ENO1* F	5’-TGGTGTCTATCGAAGATCCCTT-3’
*ENO1* R	5’-CCTTGGCGATCCTCTTTGG-3’
*TUBULIN* F	5’-TGGACTCTGTTCGCTCAGGT-3’
*TUBULIN* R	5’-TGCCTCCTTCCGTACCACAT-3’
The sequences of primers used for RT-qPCR (mouse)	
*Pcna* F	5’-TTGCACGTATATGCCGAGACC-3’
*Pcna* R	5’-GGTGAACAGGCTCATTCATCTCT-3’
*Tubulin* F	5’-CACCTGCAAGCCGGTCAAT-3’
*Tubulin* R	5’-TCCCCATGATAGGTCCCAGTG-3’
The sequences of primers used for plasmid construction.	
pCDNA3.1-ATF4 F	5’-GGGAGACCCAAGCTGGCTAGCCTCGAGG CCACCATGACCGAGATGAGCTTCCTGAA-3’
pCDNA3.1-ATF4 R	5’-GTCATGGTCTTTGTAGTCC AACTCTCTTCTTCCCCCTTG-3'
The sequences for siRNA	
*si-ATF4#1*	5’-CCAAAUAGGAGCCUCCCAUTT-3’
*si-ATF4#2*	5’*-*AUGGGAGGCUCCUAUUUGGTT*-*3'

### Cell counting Kit-8 assay

The CCK-8 assay kit (C005, USA) was obtained from TargetMOI. A 100 ul cell suspension was inoculated into a 96-well plate and incubated at 37 °C in a culture incubator with 5% CO_2_ for 24 min. Subsequently, the cells to be tested were extracted, and a 1/10 volume of CCK-8 was added directly to the cell culture medium, followed by thorough mixing and the addition of 10 ul of testing reagent. The cells were then cultured for an additional 2 min in the cell culture incubator. Post-culture, the absorption at 450 nm was measured, and cell activity was calculated.

### Ethynyl-2-deoxyuridine (EdU) staining assay

The EdU Apollo DNA in Vitro Kit (C0071S, Beyotime, China) was used to evaluate cell proliferation following the manufacturer’s instructions. Some cells were analyzed using flow cytometry with EdU staining, while others were subjected to cellular immunofluorescence staining. The EdU index (%) was calculated as the average ratio of EdU-positive cells to total cells in five randomly selected areas under a fluorescence microscope.

### Colony formation assay

HSC-1 and A431 cells were transfected with targeted shRNAs or plasmids and seeded in 6-well plates at a density of 500 cells per well. The cells were suspended in media containing 10% FBS and cultured for 14 days in a humidified atmosphere with 5% CO_2_ at 37 °C to allow for colony formation. Afterward, the colonies were fixed with 10% formaldehyde for 5 min and then stained with 0.1% crystal violet for 20 min.

### Annexin V/PI double staining

HSC-1 and A431 cells were seeded in 6-well plates and cultured for 24 min. Apoptotic cells were identified using the Annexin V/PI apoptosis detection kit (CA1012, Solarbio, China) following the manufacturer’s protocol. Briefly, the cells were resuspended in buffer, incubated with Annexin-V/PI for 10 min at room temperature, and then analyzed using flow cytometry.

### TUNEL staining

A TUNEL staining kit (G1504, China) was acquired from Servicebio. Each well of cells was treated with 50 µl of Equilibration Buffer and incubated at room temperature for 10 min. Following the removal of the Equilibration Buffer, TdT incubation buffer was added dropwise and incubated at 37 °C for 1 h. After rinsing with PBS three times, nuclear staining was performed using DAPI, and the sample was examined under a fluorescence microscope after sealing.

### Wound-healing assay

HSC-1 and A431 cells were inoculated at a density of 5 × 10^5^ cells per well in a 6-well plate. After 24 h, a scratch was created using a sterile pipette tip. The cells were then placed back in the incubator for further cultivation, and the wound healing area was observed at 0, 24, and 48 h.

### Trans-well migration and invasion assay

Cell suspensions containing 3 × 10^4^ cells per well were added with or without 50 µl of Matrigel. In the lower chamber, 500 µl of DMEM with 10% FBS was added. The plate was then incubated in a cell culture incubator for 24 h. After incubation, the upper chamber was carefully removed, cells were fixed with polymethanol for 5 min, and stained with 0.1% crystal violet for 15 min. Cells were rinsed slowly with running water and different areas were selected for cell counting.

### Western blot

The tissue or cells were fully lysed, followed by protein extraction and SDS-PAGE electrophoresis. Subsequently, the samples were transferred to a PVDF membrane using a membrane transfer instrument and blocked with 5% skim milk for 2 h. Following this, the membrane was incubated overnight at 4 °C with primary antibodies, which included METTL1 (Abcam, ab271063, 1:1000), ATF4 (Abcam, ab216839, 1:1000), PCNA (CST, 13110, 1:1000), BCL-2 (BIOSS, bs-0032R, 1:800), BAX (BIOSS, bsm-33283M, 1:1000), and *β*-Tubulin (CST, 2146, 1:2000). The visualization of target protein bands was achieved using the Chemiscope 6000 pro touch imaging system. The full length western blots are included in the supplemental material.

### Quantitative real-time polymerase chain reaction

Trizol was utilized for total RNA extraction, followed by reverse transcription using the PrimeScript RT reagent Kit (Takara Bio, Dalian, China) as per the manufacturer’s instructions. The specific primers used are listed in Table 2.

### Xenograft tumorigenesis model

Female BALB/c nude mice (Vital River, Beijing, China) aged 6 weeks were utilized to establish a subcutaneous transplanted tumor model. The BALB/c nude mouse transplanted tumor model involved inoculating animals with HSC-1 and A431 cells (5 × 10^6^) treated with shCtrl/shMETTL1 and shMETTL1+OE-Ctrl/shMETTL1+OE-ATF4, respectively. Tumor volume was monitored every 3 days, and after 30 days post-injection, the nude mice were euthanized, and all tumors were excised. The animal experiments were carried out following the protocol approved by the Animal Ethics Committee of Anhui Medical University (Approval Number: LLSC20240910).

### Immunohistochemistry

Immunohistochemistry was conducted on skin tissues following the methodology outlined in our previous study [45]. Tumor tissues were initially fixed in 10% formaldehyde for 24 h. Subsequently, paraffin embedding was carried out, and paraffin sections were generated, followed by a 2-h deparaffinization process in dimethylbenzene. Rehydration was then achieved through a series of ethanol concentrations, starting from 100% and gradually decreasing to 70%. Finally, the paraffin sections were subjected to staining with primary antibodies. The negative control group utilizes the same tissue sections as the experimental group but incubates them with PBS instead of the specific primary antibody. The remaining staining procedures are unchanged. Subsequently, drops of DAB chromogenic solution are applied to the tissue sections of both the negative control and experimental groups. Observations are conducted under a microscope until tan staining becomes evident in the experimental group, while no tan staining is observed in the negative control group. The duration of color development noted during this process is regarded as the final time for IHC color development of the tissue sections discussed in this article, and this duration will remain consistent for subsequent sections across each group. Furthermore, the results of immunohistochemical staining must be evaluated in conjunction with histomorphology to determine whether the staining indicates a true positive result rather than nonspecific staining.

### RNA-sequencing

Total RNA was extracted from HSC-1 cells treated with or without shMETTL1 using the TRIzol reagent. RNA-seq analyses were conducted by OE Biotech (Shanghai, China). Genes with a |log2FC|≥0.585 and a *p*-value and *q*-value cut-off ≤1 were classified as differentially expressed genes (DEGs). The sequence data was deposited in the NCBI Sequence Read Archive under the accession number PRJAN1102036.

### mRNA isolation and decapping

mRNA purification from total RNA was accomplished using the Dynabeads mRNA Purification Kit (61,006, Invitrogen). Subsequently, 6 µg of purified mRNA were incubated with 5 µl of Decapping Reaction Buffer (100 mM Tris-HCl pH 7.5, 1.0 M NaCl, 20 mM MgCl2, and 10 mM DTT), 1 µl of 50 mM MnCl2, 2 µl of SUPERase-In RNase Inhibitor (AM2696, Thermo Fisher Scientific), and 8 µl of Tobacco decapping enzyme (94, Enzymax) in a final volume of 50 µl.

### Dot blot

RNA was extracted and sample concentrations were determined. Subsequently, the RNA was diluted to 100 ng/µl and 200 ng/µl. Denaturation of the RNA was achieved by heating at 95 °C for 5 min, and 1 µl of each concentration was then applied onto a nitrocellulose membrane. Following drying, UV cross-linking was performed for 5 min. The membrane was then incubated in 10% skim milk for 1 h, washed 3 times with TBST for 5 min each, and primary anti-m7G (Abcam, ab300740, 1:1000), was applied overnight at 4 °C. Subsequent washing was performed 3 times with TBST for 5 min each, followed by incubation with the secondary antibody at room temperature for 1 h. Finally, the membrane was washed 5 times with TBST for 5 min each.

### MeRIP-qPCR

The utilized protocol for MeRIP was previously outlined [46]. In brief, the MeRIP-qPCR assay was conducted in HSC-1 and A431 cells using the Magna RIP Kit (17-700, Millipore, MA) according to the manufacturer’s instructions. A total of 2 × 10^7^ cells were lysed, and RNA was enriched using either 10 μg of anti-M7G antibody or IgG-loaded beads (#16-663, Sigma-Aldrich). The enriched RNA was then purified and assessed using qRT-PCR.

### RNA stability assay

Cells were cultured in 6-well plates and transfected with the specified constructs as outlined above. Following a 24-h transfection period, the cells were exposed to actinomycin D (Act D, 10 μg/mL, Cat# GC16866, GLPBIO) for 0, 3, or 6 min before being collected. Total RNA was then isolated for qRT-PCR analysis.

### Seahorse analysis

Cells were seeded in a 96-well Seahorse XF Cell Culture Microplate (2 × 10^4^ cells/well, Agilent, USA). Following overnight incubation, the culture medium was replaced as per the instructions provided in the Agilent Seahorse XF Cell Mito Stress Test Kit and the XF Glycolysis Stress Test Kit. Subsequently, the microplate was incubated in a non-CO_2_ incubator at 37 °C for 1 h prior to measuring the oxygen consumption rate (OCR) and extracellular acidification rate (ECAR) in the XF Analyzer, following the procedural guidelines.

### JC-1 assay

Detection of the mitochondrial membrane potential was performed using a JC-1 kit purchased from MKBio (MX3203, China) according to the manufacturer’s instructions. A total of 10,000 cells/sample were analyzed to assess their fluorescence intensity using a BD FACS Celesta instrument.

### Detection of mitochondrial mass

Mitochondrial mass was measured using MitoTracker Green FM (MX4309, MKBio, China). A total of 10,000 cells per sample were analyzed in the FITC channel using a BD FACS Celesta instrument.

### Glucose level detection

Glucose uptake was assessed using the Glucose Assay Kit (A154-2-1, Nanjing JianCheng Bioengineering Institute). After allowing the cells to adhere to the culture surface, 2 × 10^4^ cells were collected in a centrifuge tube and centrifuged at 1000 rpm. The supernatant was discarded, and the cells were washed twice with PBS. To prepare the homogenate solution for subsequent experiments, 0.3 mL of PBS was added for homogenization, followed by sonication in an ice water bath. The reagents were added sequentially according to the manufacturer’s instructions, and a microplate reader was used to measure the glucose content in the culture supernatant. After 24 h of culture, the glucose content in the cell culture medium was measured again. The initial glucose level was subtracted from the subsequent measurement to determine the amount of glucose taken up by the cells.

### ATP production assay

ATP production was measured by ATP production kit (A095-1-1, Nanjing JianCheng Bioengineering Institute). After allowing the cells to adhere to the culture surface and incubating for 24 h, 2 × 10^6^ cells were collected and centrifuged at 1000 rpm. The collected cells were then added to 300 µL of cold double-distilled water for homogenization and disruption. Subsequently, the cell suspension was heated in a boiling water bath for 10 min, after which it was removed and mixed thoroughly for extraction over a duration of 1 min. The absorbance values of each tube were measured at 636 nm using the corresponding reagents, following the manufacturer’s instructions.

### Lactate level detection

The lactate level of cells was detected by Lactic Acid assay kit (A019-2-1, Nanjing JianCheng Bioengineering Institute). Collect the supernatant from HSC-1 and A431 cells. Sequentially add distilled water, standard solution, enzyme working solution, test sample, and colorimetric solution to a 96-well plate, following the provided instructions. Mix the contents thoroughly, then incubate in a water bath at 37 °C for 10 min. Finally, add the termination solution dropwise and conduct detection using a microplate reader set to 530 nm.

### Luciferase reporter assay

HSC-1 or A431 cells were seeded in six-well plates and transfected with the pSI-CHECK2 luciferase vector (Hanbio Biotechnology, China) fused with or without the wild-type or mutated ATF4-5’UTR. All cells were harvested 48 h after transfection, and the firefly luciferase and Renilla luciferase activities in each well calculated by a dual-luciferase reporter assay system. The relative ratio of firefly luciferase activity to Renilla luciferase activity was determined.

HSC-1 or A431 cells were co-transfected with OE-Ctrl, OE METTL1+siCtrl or OE METTL1+siATF4 and pGL3-HK2 promoter. pGL3-HK2 promoter was purchased from Hanbio Biotechnology (Shanghai, China). After 48 h of transfection, the cells were transferred to 96-well plates and the luciferase activity was measured with the dual-luciferase reporter assay system, following manufacturer’s instructions.

### Statistical analysis

Data are means ± SEM. Statistical significance between two groups was determined using a two-tailed Student’s *t* test. For multiple comparisons, a two-way ANOVA followed by Tukey’s post hoc test was conducted to determine statistical significance. *P < 0.05, **P < 0.01, ***P < 0.001. Data are representative of three independent experiments.

## Supplementary information

Supplementary figures

Full and uncropped western blots

## Data Availability

The data that support the findings of this study are available on request from the corresponding authors upon reasonable request.
