# Supplementary material for: METTL1 coordinates cutaneous squamous cell carcinoma progression via the m7G modification of the ATF4 mRNA
Source: Cell Death Discov. 2025 Jan 27;11:27. doi: 10.1038/s41420-025-02304-3 (PMC11772585; doi:10.1038/s41420-025-02304-3)
Supplement: Supplementary file 1 — Supplementary figures [file 41420_2025_2304_MOESM1_ESM.pdf]

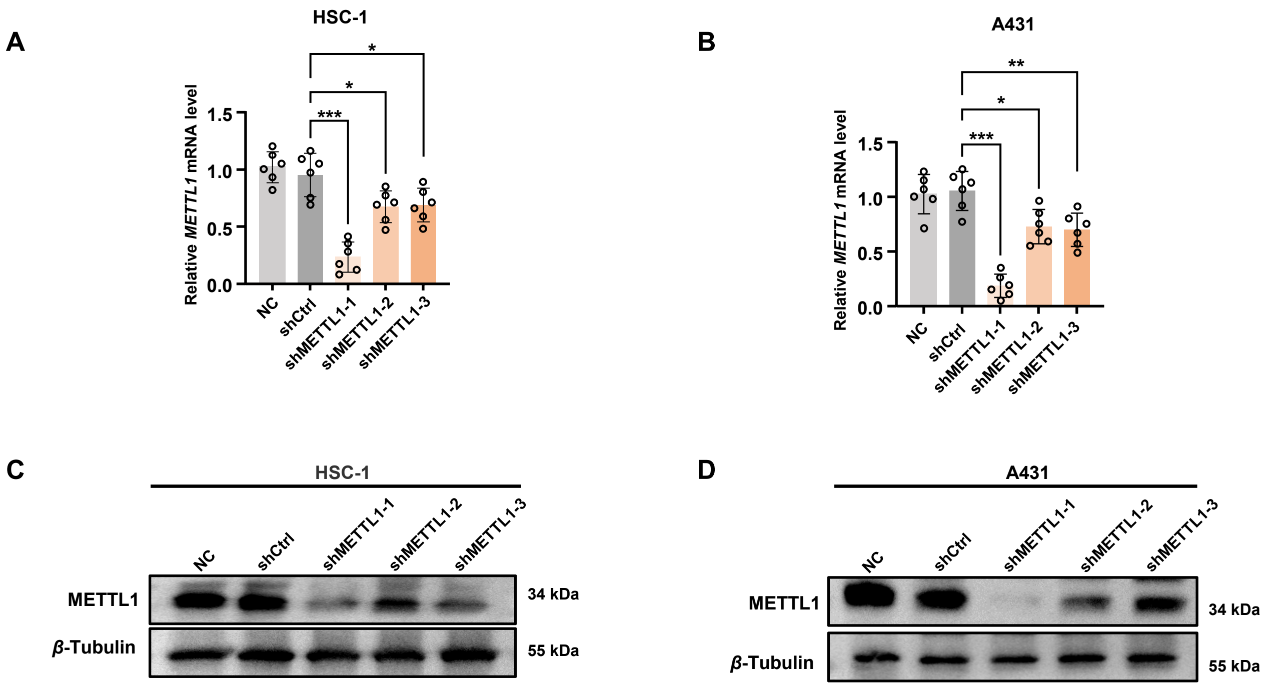

Figure S1. METTL1 knockdown was successfully achieved in cSCC cells. (A-B) The mRNA levels of *METTL1* were assessed in HSC-1 or A431 cells transfected with shMETTL1-1, shMETTL1-2, and shMETTL1-3. **(C-D)** The protein levels of METTL1 were examined in HSC-1 or A431 cells transfected with shMETTL1-1, shMETTL1-2, and shMETTL1-3. Data are shown as mean ± SEM. n=6. *P<0.05, ***P<0.001.

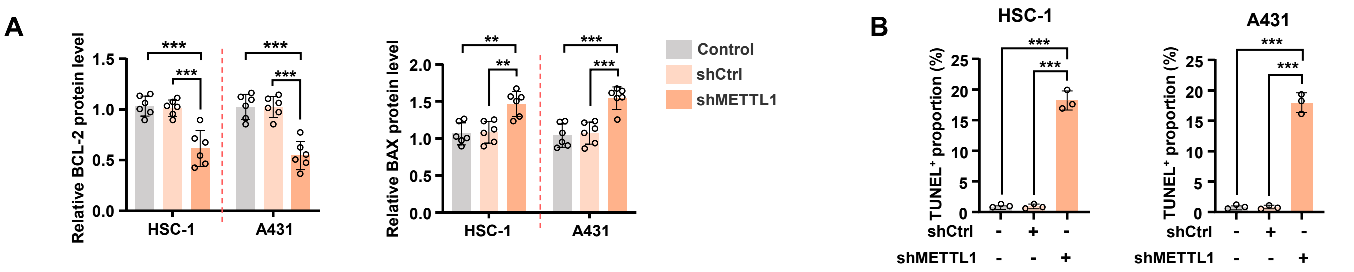

Figure S2. Inhibiting METTL1 promoted cSCC cells death. (A) The quantitative data in **Fig. 2I**. n=6. **(B)** The quantitative data in **Fig. 2K-L**. n=3. Data are shown as mean ± SEM. ***P<0.001.

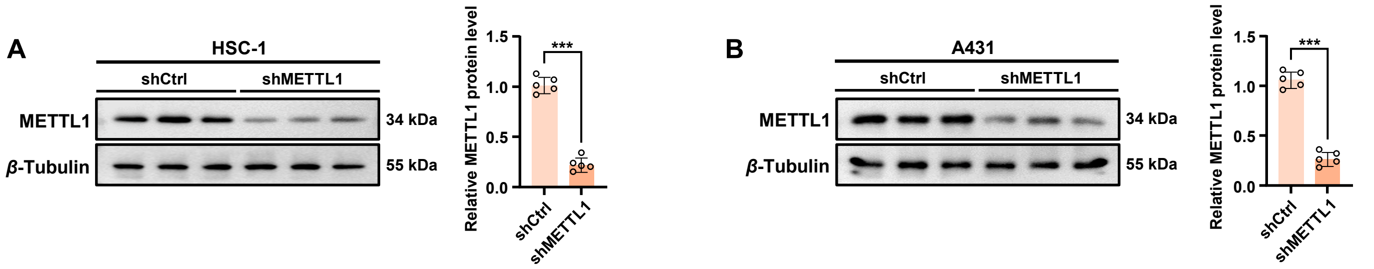

Figure S3. METTL1 levels were inhibited in tumor tissues upon METTL1 knockdown. (A-B) The protein levels of METTL1 in tumor tissues were detected by western blot. *β*-Tubulin was used for the normalization control. The relative protein level was calculated. Data are shown as mean ± SEM. n=5. ***P<0.001.

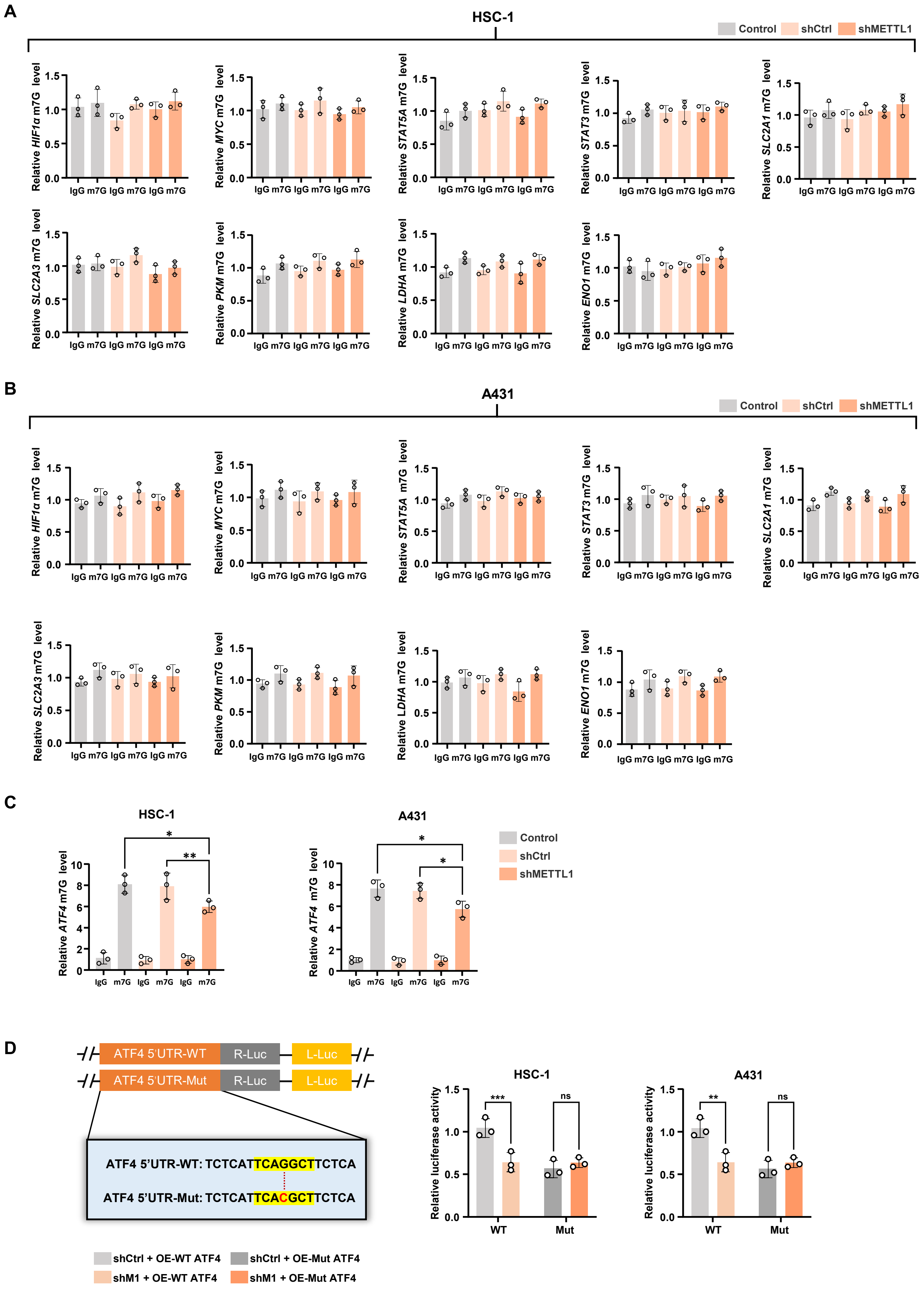

Figure S4. METTL1 did not mediate m7G modification of *HIF1α*, *MYC*, *STAT5A*, *STAT3*, *SLC2A1*, *SLC2A3*, *PKM*, *LDHA*, and *ENO1* mRNA. (A-B) MeRIP-qPCR analysis revealed no significant m7G modification of the mentioned mRNA in HSC-1 and A431 cell lines (n=3). (C) Suppressed m7G methylated decapped *ATF4* mRNA in METTL1-knockdown cells was detected by MeRIP-qPCR (n=3). **(D)** Wild-type (WT) and mutant (Mut) ATF4 were cloned into psiCHECK2 reporter vectors. The luciferase activities of *ATF4* were measured in HSC-1 or A431 cells with or without METTL1 knockdown (n=3). Data are shown as mean ± SEM.

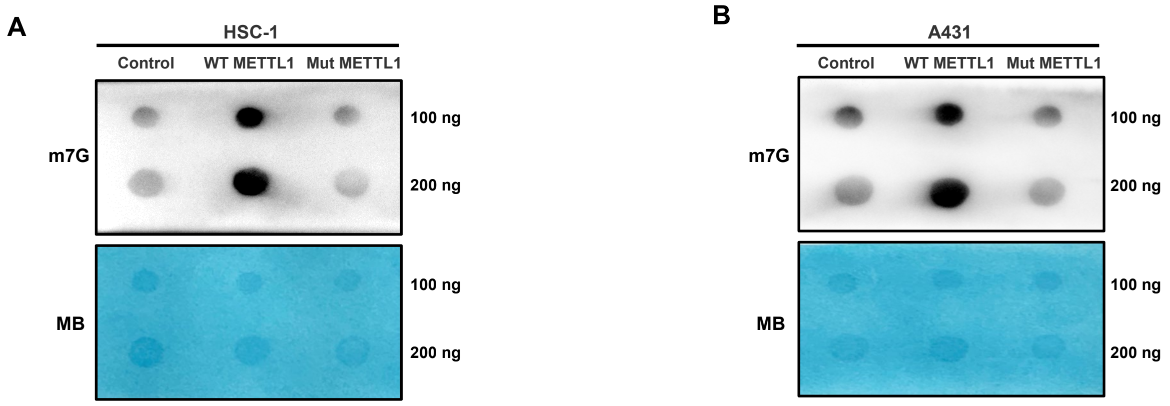

Figure S5. Increased WT METTL1 significantly elevated m7G methylation levels, rather Mut METTL1. (A-B) The m7G methylation level in each group were detected by dot blot assay (n=3).

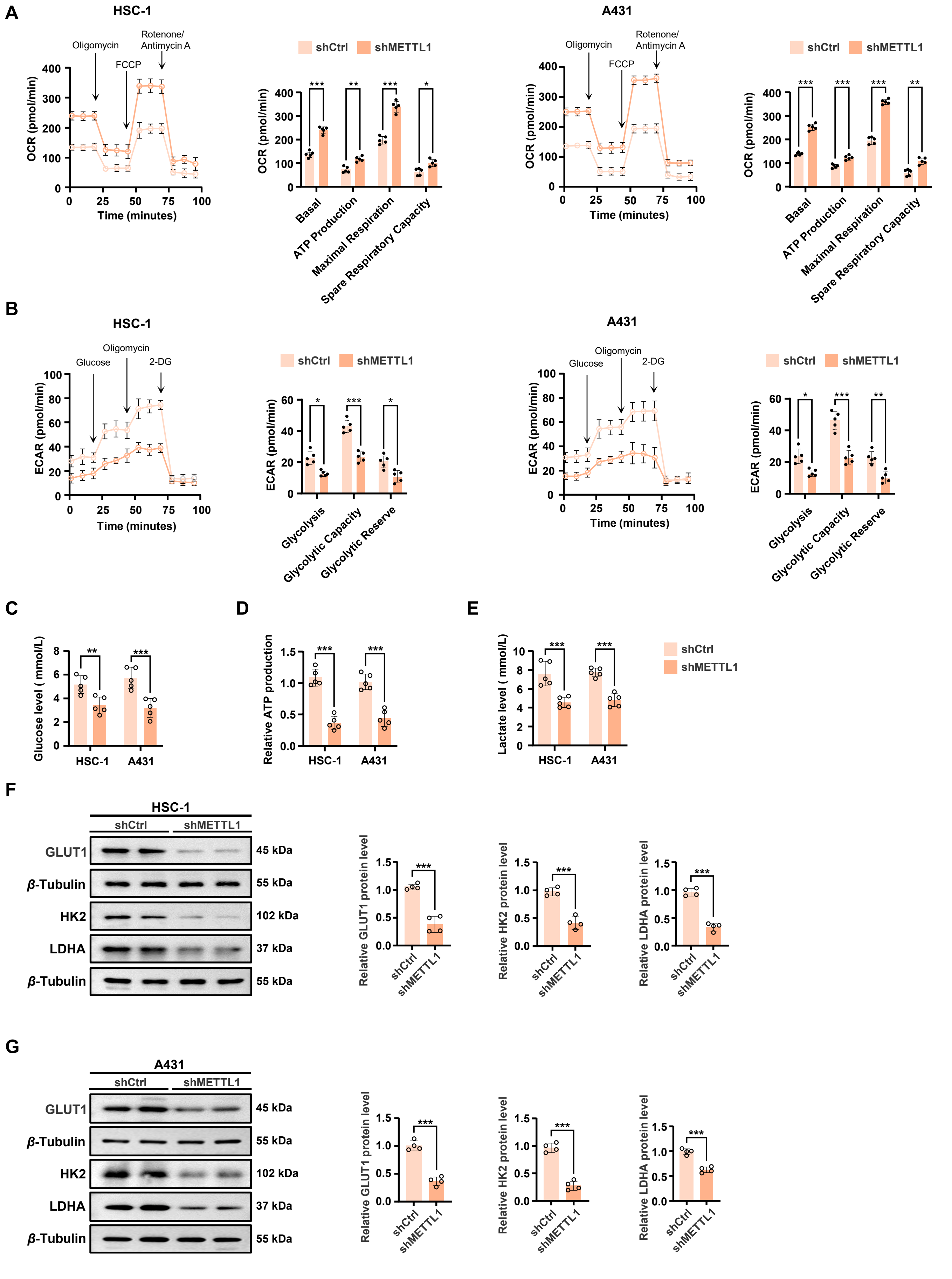

Figure S6. METTL1 knockdown inhibited glycolysis but promoted oxidative phosphorylation in HSC-1 and A431 cells. (A-B) Metabolic phenotypes of HSC-1 and A431 cells with or without METTL1 knockdown were determined by the both OCR and ECAR assays (n=5). **(C)** Glucose uptake in HSC-1 and A431 cells were detected glucose assay (n=5). **(D)** The ATP production was evaluated through ATP assay kit (n=5). **(E)** Lactate levels in the medium were assessed (n=5). **(F-G)** The protein levels of GLUT1, HK2 and LDHA were detected by western blot in HSC-1 and A431 cells (n=4). *β*-Tubulin was used for the normalization control. Relative protein levels were calculated. Data are shown as mean ± SEM. **P*<0.05, ***P*<0.01, ****P*<0.001.

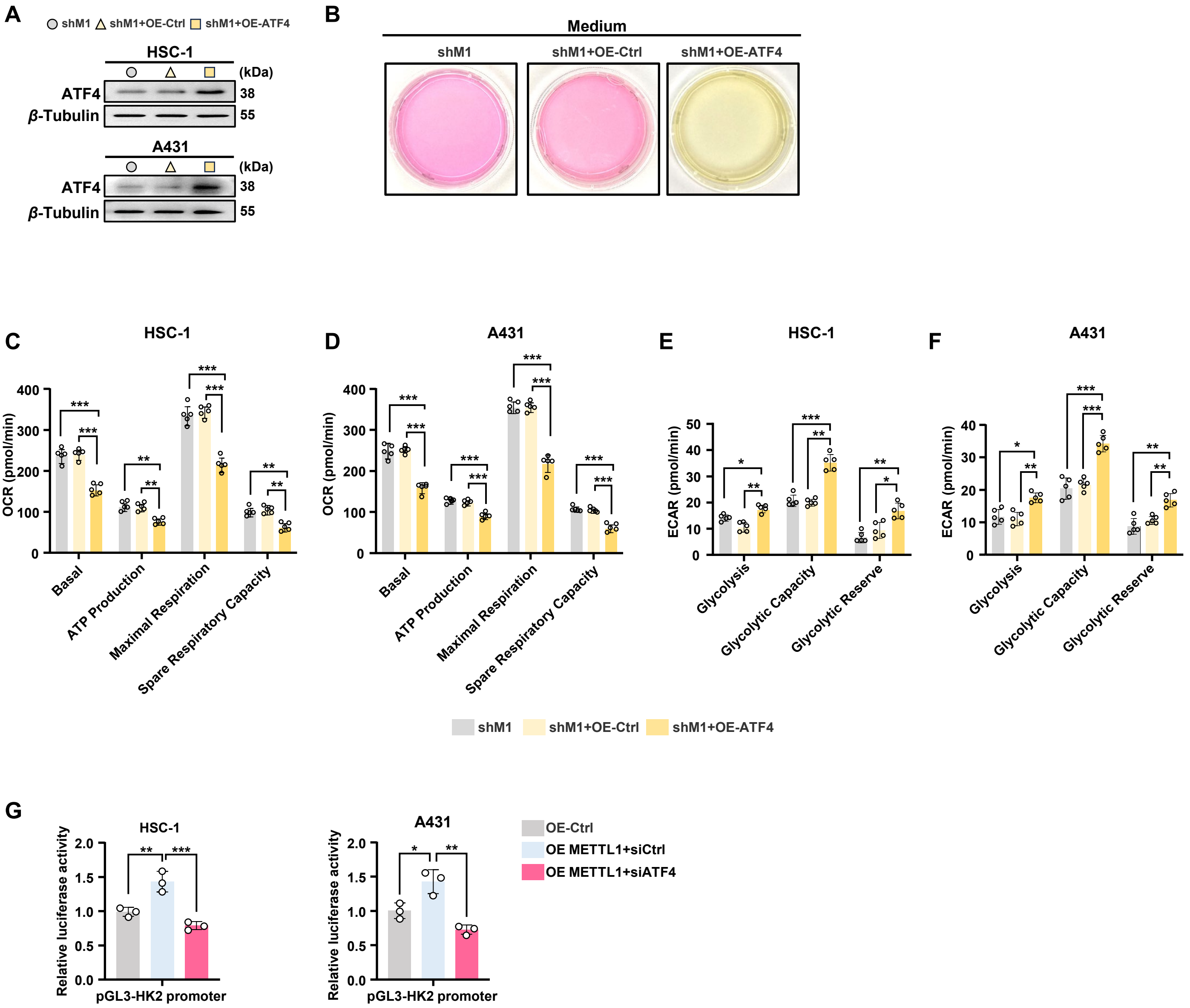

Figure S7. Upregulation of ATF4 promoted glycolysis in METTL1-knockdown cSCC cells. (A) The protein levels of ATF4 in each group were detected by western blot (n=3). **(B)** Representative images of cell culture supernatant. **(C-D)** The quantitative data in **Fig. 6A**. n=5. **(E-F)** The quantitative data in **Fig. 6B.** n=5**. (G)** Luciferase reporter assays were conducted to measure the luciferase activity of *HK2* in cells overexpressing METTL1, both with and without siATF4 transfection (n=3). Data are shown as mean ± SEM. **P*<0.05, ***P*<0.01, ****P*<0.001.

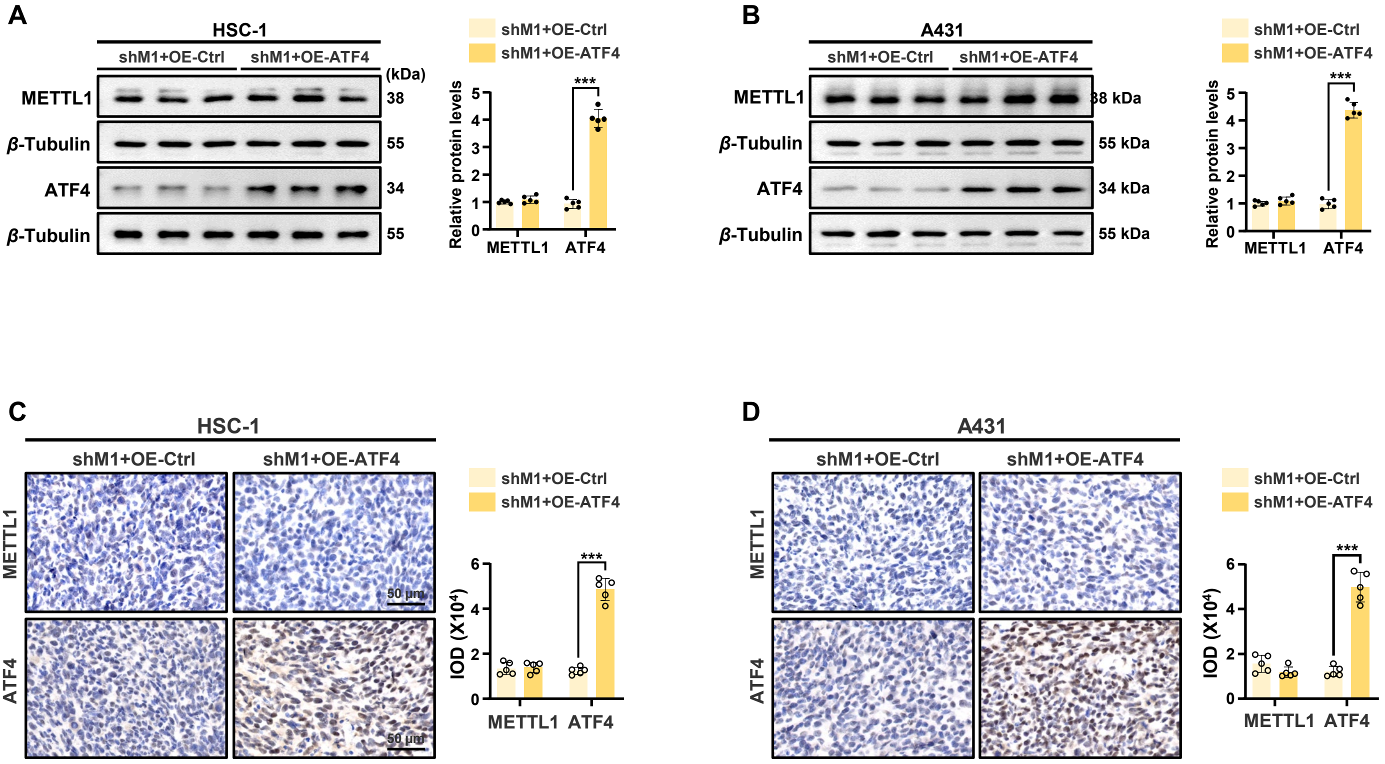

Figure S8. ATF4 expressions were increased in tumor tissues of nude mice after injections of METTL1-knockdown HSC-1 and A431 cells transfected with ATF4-overexpression plasmid. (A-B) The protein levels of METTL1 and ATF4 were measured by western blot (n=5). *β*-Tubulin was used for the normalization control. Relative protein levels were calculated. **(C-D)** The protein levels of METTL1 and ATF4 were measured by IHC (n=5). The IOD for each sample was calculated. Data are shown as mean ± SEM. ****P*<0.001.
