# Supplementary figures and images for: METTL1 coordinates cutaneous squamous cell carcinoma progression via the m7G modification of the ATF4 mRNA
Source: Cell Death Discov. 2025 Jan 27;11:27. doi: 10.1038/s41420-025-02304-3 (PMC11772585; doi:10.1038/s41420-025-02304-3)

Fig. 1D

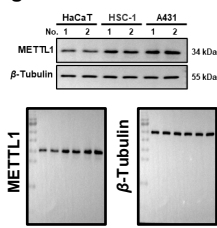

Fig. 2A

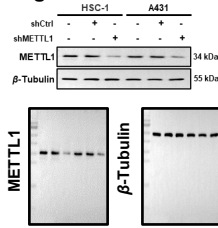

Fig. 2I

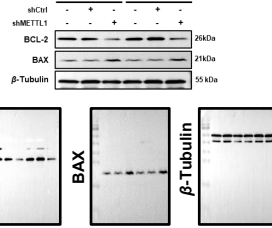

Fig. 4F

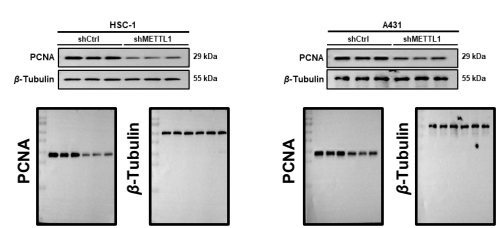

Fig. 5G

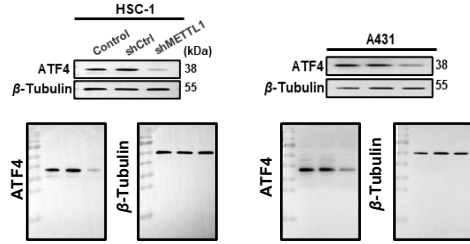

Fig. 5J

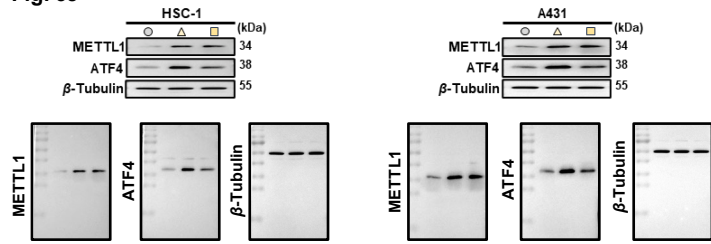

Fig. 6F

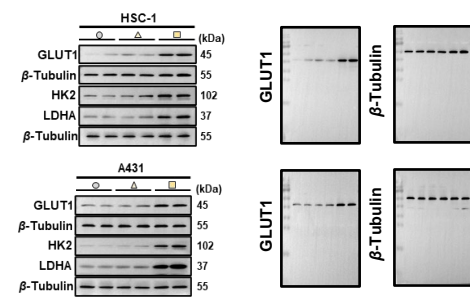

Fig. 7F

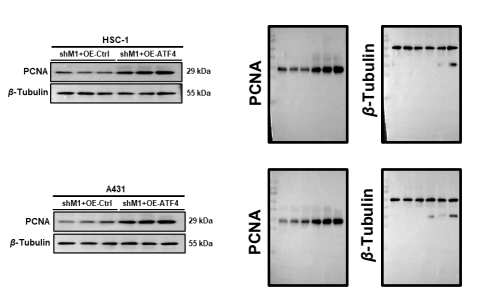

Fig. S1C

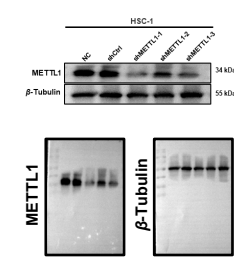

Fig. S1D

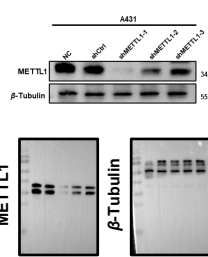

Fig. S6F

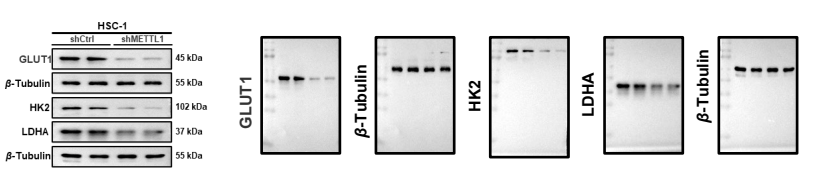

Fig. S6G

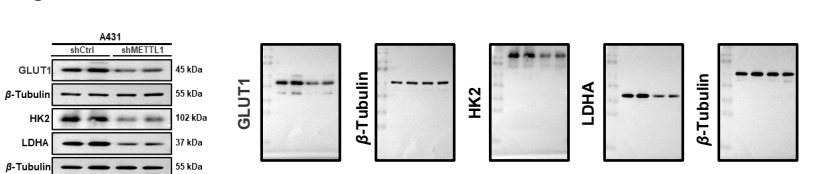

Fig. S3A

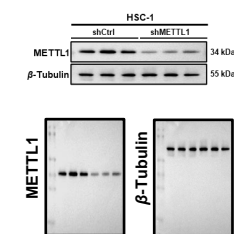

Fig. S3B

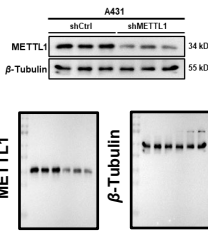

Fig. S7A

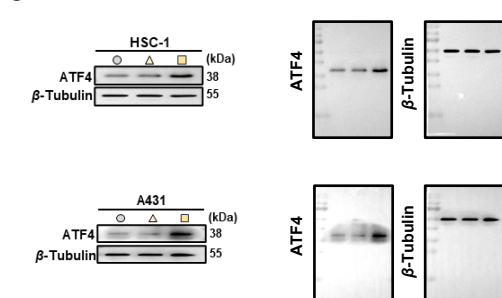

Fig. S8A

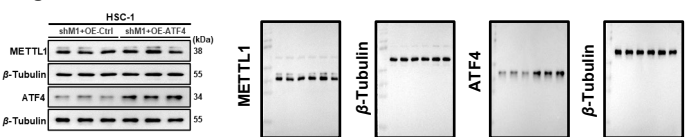

Fig. S8B

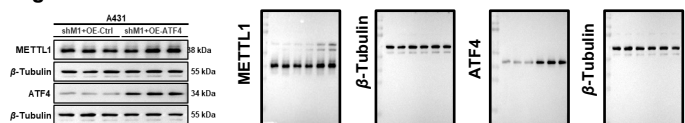

Supplement: Supplementary file 2 — Full and uncropped western blots [file 41420_2025_2304_MOESM2_ESM.pdf]
